# The Ketamine Trial for Acute Suicidality (KETA): Study Protocol of a Double‐Blind Randomized Placebo‐Controlled Superiority Trial on Intranasal Racemic Ketamine Compared to the Active Placebo Intranasal Midazolam as Treatment for Acute Suicidality

**DOI:** 10.1002/mpr.70044

**Published:** 2025-11-19

**Authors:** Jurriaan F. M. Strous, Gijs H. J. Roelandt, Jens H. van Dalfsen, Jeanine Kamphuis, Radboud M. Marijnissen, Robert A. Schoevers

**Affiliations:** ^1^ Department of Psychiatry University of Groningen University Medical Center Groningen Groningen the Netherlands; ^2^ Lentis Mental Health Care Groningen the Netherlands

**Keywords:** ketamine, randomized controlled trial, suicidality

## Abstract

**Background:**

Suicidality is a transdiagnostic entity in patients with and without psychiatric disorders. Ketamine is a novel treatment for treatment‐resistant depression with favorable effects on related suicidality in this population. Little is known about the effects of ketamine on suicidality as a distinct phenomenon.

**Objective:**

To assess whether a dose of 75 mg intranasal (IN) ketamine reduces acute suicidality relative to a 4 mg intranasal dose of the active placebo midazolam, 180 min after administration.

**Methods/design:**

A double‐blind randomized placebo‐controlled trial (*N* = 100) will assess the efficacy of fixed‐dose IN racemic ketamine in patients presenting with acute suicidality, regardless of psychiatric diagnosis. Participants receive a single IN dose of either racemic ketamine (75 mg) or midazolam (4 mg) along with treatment as usual. The primary outcome is the reduction in suicidal ideation as measured by the Beck Scale for Suicide Ideation (BSSI) at 180 min. Secondary outcomes include depression severity with the Montgomery Åsberg Depression Rating Scale (MADRS) and tolerability with the Systematic Assessment for Treatment Emerging Effects (SAFTEE) as well as blood‐based and neuroimaging markers.

**Discussion:**

This study design considers key aspects such as patient selection, ketamine formulation, clinical management, and the follow‐up time points. Potential risks, limitations, and future considerations are additionally discussed.

**Trial Registration:**

EudraCT 2020‐002905‐24, registered 6 October 2021

**Abbreviations**
ACC: anterior cingulate cortex
AMDP‐SS: Association for Methodology and Documentation in Psychiatry Somatic Signs
ANCOVA: analysis of covariance
ANOVA: analysis of variance
BA10: Brodmann Area 10
BAC: blood alcohol concentration
BDNF: brain derived neurotrophic factor
BSSI: Beck Scale for Suicide Ideation
CGI: Clinical Global Impression
CPRS: Comprehensive Psychopathological Rating Scale
CRO: Clinical Research Office
CTQ‐SF: Childhood Trauma Questionnaire Short Form
DSM‐5: Diagnostic and Statistical Manual of Mental Disorders, Fifth Edition
DSMB: Data Safety Monitoring Board
DTI: diffusion tensor imaging
ELISA: enzyme‐linked immunosorbent assay
FA: fractional anisotropy
fMRI: functional magnetic resonance imaging
GHB: gamma‐hydroxybutyrate
GI: general inquiry
HDRS: Hamilton Depression Rating Scale
ICA: independent component analysis
IN: intranasal
IRB: Institutional Review Board
IV: intravenous
KETA: Ketamine Trial for Acute suicidality
KNMG: Koninklijke Nederlandse Maatschappij tot bevordering der Geneeskunst (Royal Dutch Medical Association)
MADRS: Montgomery Åsberg Depression Rating Scale
MDD: major depressive disorder
MINI: Mini Neuropsychiatric Interview
MRI: magnetic resonance imaging
MRS: MR‐based spectroscopy
NMDA: N‐Methyl‐D‐aspartate
PO: oral
SAE: serious adverse event
SAFTEE: Systematic Assessment for Treatment Emerging Effects
SI: suicidal ideation
SPC: summary of product characteristics
SUSAR: suspected unexpected serious adverse reaction
*T*_max_
: time to maximum concentration
UMCG: University Medical Center Groningen
WHO: World Health Organization
^1^H‐MRS: proton magnetic resonance imaging

## 1 Background

According to the World Health Organization (WHO) more than 700,000 people die by suicide each year. For every successful suicide there are 20 failed attempts, exerting a large impact on patients, family, and the healthcare system (World Health Organization 2024). In 2021, the worldwide suicide incidence varied from 0.5 per 100,000 (Saint Vincent and the Grenadines) to 28.7 per 100,000 (Lesotho) (World Health Organization 2024). In the Netherlands, the average suicide rate was 11.2 per 100,000 per year in 2024 (113 2025), with a reported increase of 30% between 2008 and 2022 (113 2023a). This is in contrast with the steadily decreasing trend worldwide: from 755,460 in 2008 to 718,033 in 2021 (Our World in Data 2025), while the world population grew from 6.81 billion in 2008 to 7.76 billion in 2021 (Our World In Data). Although there appears to be a downward trend globally, the absolute suicide rate remains high, and suicide currently represents the leading cause of death of persons under 30 in the Netherlands (113 2023b), the fourth leading cause of death in 15–29‐year‐olds worldwide (World Health Organization, 2023), and the second leading cause of death for people aged 10‐14 and 20‐34 in the United States (Centers for Disease Control 2024). This indicates that little progress has been made in the prevention and treatment of suicidality emphasizing the need for more effective interventions.

Although suicidality is a prevalent medical emergency with extensive societal impact, it is currently not listed as a separate diagnostic entity. In the Diagnostic and Statistical Manual of mental disorders (DSM‐5) (Diagnostic and Statistic Manual 5 2013) suicidal behavior is classified as a symptom of major depression or borderline personality disorder, as well as in relation to other disorders such as schizophrenia or substance use disorder (113 2023b). However, suicide also occurs independently of these disorders. Approximately 10% of suicide victims had no diagnosable psychiatric disorder (Aleman and Denys 2014; Oquendo et al. 2008) and a network analysis showed that the symptom cluster for acute suicidal affective disorder, a newly proposed diagnosis for suicidality, was markedly distinct from symptom clusters of anxiety and depressive disorders (Rogers et al. 2019). For this reason, it has been proposed that suicidality should be considered and diagnosed as a distinct disorder, possibly leveraging more knowledge about its nature and treatability (Fehling and Selby 2021).

Acute suicidality is usually treated by means of a supportive and/or psychotherapeutic intervention, in which family or other members of a person's social network are involved when possible. Such treatment may take place in an emergency department setting of either a general hospital or a mental care institution. A common strategy is to assess patients at risk for suicide and follow them up repeatedly. This intervention has been shown to decrease suicidal acts (Luxton et al. 2013). In addition, follow‐up up by phone calls or sending postcards has been studied. A transdiagnostic meta‐analysis showed that after active contact and follow‐up the number of suicide attempts within the 12 months after the start of the intervention decreased significantly (Inagaki et al. 2019). Evidence further suggests that the number of suicides and suicide attempts decreases immediately following cognitive behavioral therapy or dialectical behavioral therapy specifically targeted at subgroups, primarily patients with borderline personality disorder (Meerwijk et al. 2016). Although these psychological interventions are partly efficacious, and may forestall suicides, their ability to diminish acute suicidality within hours is limited. Hence, there is a need for more effective and faster‐acting treatment options.

Given its fast and strong antidepressant effect, ketamine may act synergistically with either direct or indirect psychological interventions in the treatment of suicidality. This N‐methyl‐D‐aspartate (NMDA) receptor antagonist has primarily been investigated in depressed patients for its potential antidepressant effects. In several randomized controlled trials in patients with treatment resistant unipolar or bipolar depression, either a single or repeated subanaesthetic dose(s) of intravenous (IV), intranasal (IN), or oral (PO) (es)ketamine have been shown to exert rapid antidepressant effects with minimal adverse effects (aan het Rot et al. 2010; Price et al. 2009; Lapidus et al. 2014; Singh et al. 2016; Smith‐Apeldoorn, Vischjager, et al. 2022). Building on the established antidepressant efficacy, several clinical trials investigated the influence of ketamine on suicidality in depressed subjects (Murrough et al. 2015; Grunebaum et al. 2018). In these populations ketamine yielded a significantly greater reduction in suicidality compared to placebo (Singh et al. 2016; Smith‐Apeldoorn, Vischjager, et al. 2022).

Elaborating on the findings in depression, recent studies have evaluated the efficacy of ketamine for suicidality as a transdiagnostic entity. Domany and McCullumsmith (2022) found a statistically insignificant reduction in suicidal ideation (SI) 4 hours after ketamine administration in patients presenting with suicidality at an emergency department (Domany and McCullumsmith 2022). In line with these findings, Abbar et al. (2022) reported a significant reduction of SI with 63% of the included subjects achieving remission 3 days after receiving ketamine (Abbar et al. 2022). Interestingly, the effects were strongest in the bipolar depression group and did not reach significance in patients with major depressive disorder or other psychiatric disorders (Murrough et al. 2015; Grunebaum et al. 2018). While these initial studies demonstrate that ketamine may act as a transdiagnostic treatment for acute suicidality, it remains uncertain whether ketamine yields an antisuicidal effect independent from its antidepressant effect (Apeldoorn et al. 2017) and it is unclear which subgroups of patients may benefit most from the presumed antisuicidal properties of ketamine.

The mechanisms underlying the antisuicidal effects of ketamine are unknown. A recent meta‐analysis showed that brain derived neurotrophic factor (BDNF) may play a role in ketamine's antidepressant efficacy, since a longitudinal analysis showed that ketamine responders, but not non‐responders, had significant increases in BDNF measured in serum or plasma (Medeiros et al. 2022). Several groups demonstrated that the antidepressant response following ketamine infusion in mice is BDNF dependent as BDNF knockout mice did not respond to ketamine treatment (Autry et al. 2011; Zanos and Gould 2018). These findings indicate that BDNF might also play a role in the antisuicidal effects of ketamine. In addition to research on BDNF, functional brain alterations following ketamine treatment may contribute to the presumed antisuicidal efficacy (Zavaliangos‐Petropulu et al. 2023). Two recent studies showed neuroplastic changes after ketamine infusion in the left Brodmann Area 10 (BA10), the left amygdala, and the left and right hippocampus. Also, increased connectivity between prefrontal and limbic regions was observed. One of the studies found this result by assessing microstructure changes by diffusion tensor imaging (DTI) (Kopelman et al. 2023) and the other by revealing altered resting state and mood induced state connectivity patterns (Rengasamy et al. 2024). Also, imaging studies have been able to reveal glutamatergic changes in the hippocampus and the anterior cingulate cortex (ACC) (Stone et al. 2012), which are presumed to contribute to enhanced synaptic plasticity.

The clinical trial described in this research protocol will investigate whether a single dose of IN ketamine is more effective than an active placebo in reducing acute suicidal ideation and behavior, irrespective of the specific underlying psychiatric diagnosis. Subjects with acute suicidality (*N* = 100) will be randomized in a double‐blind manner to receive a single administration of either IN racemic ketamine (75 mg) or IN midazolam (4 mg). Patients will be monitored for suicidal ideation (Beck Scale for Suicide Ideation, (BSSI)) (Beck et al. 1979), depressive symptoms (Montgomery Åsberg Depression Rating Scale (MADRS)) (Montgomery 1979), side effects (Systematic Assessment for Treatment Emergent Effects (SAFTEE)) (Levine and Schooler 1986) and overall disease severity and change (Clinical Global Impression (CGI)) (Guy 1976) before and after treatment with the reduction in suicidality at 180 min after administration of IN ketamine compared to IN midazolam as the primary endpoint. We believe using an active placebo presents a large advantage compared to studies performed with a saline placebo, since blinding conditions will be better and, hence, the placebo effect will be diminished, offering a more truthful insight in ketamine's real antisuicidal efficacy. To evaluate potential underlying mechanisms, we perform functional magnetic resonance imaging (fMRI) in order to evaluate connectivity patterns and glutamate release, we collect blood samples for the evaluation of biomarkers, such as BDNF, for ketamine's potential antisuicidal effect. Taken together, this randomized controlled trial with active placebo aims to provide insight in the transdiagnostic antisuicidal effect of ketamine, including an evaluation of potential neurobiological mechanisms. The article concludes with a discussion of the various considerations and dilemmas that were addressed in the design of the study as well as the strengths and limitations.

## 2 Methods

### 2.1 Study Design

The current study comprises a multicenter double‐blind, randomized, placebo‐controlled trial to evaluate the efficacy of IN racemic ketamine relative to midazolam for the treatment of acute suicidality. Study participants (*N* = 100) will be allocated at random (1:1) to receive one IN dose of either ketamine (*n* = 50) or midazolam (*n* = 50) as an active placebo. Randomization is done through ALEA (FormsVision) by an independent randomization professional. The allocation concealment will be ensured since it will only be released after the potential subject has been enrolled in the trial. The pharmacy will not release the study drug allocation to the research team. Both researchers and subjects are blinded. In case an emergency occurs which necessitates knowledge if either ketamine or midazolam has been administered, the research pharmacy can provide this data. Blinding is tested by asking participants and researchers involved in data collection in which study arm they suspected they were at the end of follow‐up and comparing this to the allocation list after unblinding.

### 2.2 Study Population

The study population (*N* = 100) comprises patients aged 18–70 years who present with acute suicidality at one of the participating sites. Participants will be recruited from two locations in Groningen, the Netherlands: the University Medical Center Groningen (UMCG), a large academic hospital with in‐ and outpatient psychiatric facilities and a general emergency department with a psychiatry emergency facility, and Lentis, a regional provider of specialized mental healthcare. Subjects can be recruited through various routes. Patients may visit or be brought to the emergency department of the UMCG, or their general practitioner can arrange an appointment at the psychiatric emergency department at Lentis. Besides these allocated patients, patients that are already receiving care at the UMCG or Lentis may become eligible for participation when they experience acute suicidality. Also, patients who are treated elsewhere may be referred for participation in the study.

### 2.3 Recruitment

The first subject has been recruited in October 2022, with an average enrollment rate of two to three participants per month. At the onset of recruitment, the expected completion date was 2026. However, the recruitment phase has later been prematurely concluded following advice of the DSMB due to futility at the 50% interim analysis.

#### 2.3.1 Eligibility

An overview of in‐ and exclusion criteria for the study is provided in Table 1. Acute suicidality is defined as having had a subjective increase in suicidal ideation or behavior, including suicide attempts, in the last 72 h (96 h before the actual administration of the study drug), with a BSSI score of seven or above. This BSSI cut‐off score is corroborated by previous studies. In one ketamine study with 60 depressed subjects the mean BSSI‐baseline score was 5 (Ballard et al. 2015). In a study in which psychotic subjects were included after a suicide attempt, the mean BSSI score was 10.21 (Pinninti et al. 2002). Participation in the MRI part of the study and storage of blood in the biobank are optional, eligibility and the informed consent procedure for these ancillary options are performed separately.

**TABLE 1:** In‐ and exclusion criteria.

Inclusion criteria	
Acute suicidality	Suicidal thoughts and/or behavior have subjectively increased at any time point during the last 96 h before the hypothetical administration of ketamine/midazolam
Suicidal ideation	BSSI‐score ≥ 7
Age	18–70 years
Exclusion criteria	
Study participation	Earlier participation in this study
Psychiatric diagnosis:	Psychosis (as a primary diagnosis) (depression with psychotic features will not be an exclusion criterion per se), schizophrenia or another primary psychotic disorder
Addiction	History of phencyclidine (PCP)‐ or ketamine addiction
Drug use	Being under influence of gamma‐hydroxybutyrate (GHB). Substance abuse in the (recent) history is not an exclusion criterion per se (with the exception of current GHB‐intoxication and a high blood alcohol concentration, and intoxications leading to medical unstable conditions). Use of GHB will be assessed by asking the participant, since urinary analysis is relatively unreliable, and waiting for results of the blood test will, given the acute nature of this study, be too time consuming.
Alcohol use	A blood alcohol concentration (BAC) of > 0.05%
Medical conditions	Clinically significant and unstable infectious, immunological, neurological cardiovascular, gastro‐intestinal, pulmonary, renal, ophthalmological (glaucoma), hepatic, endocrine or haematological disorder, a myocardial infarction, micturition problems or a complex surgical problem that needs immediate attention.
Contra‐indication	Presence of any contra‐indication for ketamine use, such as severe high blood pressure, a recent myocardial infarction or relevant cardiac problems, severe thyroid problems, severe liver problems, severe kidney problems, epilepsy and increased intracranial pressure, a known hypersensitivity for ketamine.
Medication use	Concomitant use of a monoamino oxidase inhibitor (MAO‐I). Concomitant use of a potent CYP3A4 inhibitor, such as clarithromycin, erythromycin, diltiazem, itraconazole, ketoconazole, ritonavir, verapamil, goldenseal and grapefruit, concomitant use of a potent CYP3A4 inducer such as phenytoin, rifampicin, St. John's Wort and glucocorticoids.
Severe nose congestion or nasal polyps	
Pregnancy or giving breastfeeding	
Women in the reproductive age, who are heterosexually active, using unreliable contraception	
Being unable to answer the questionnaires	
Legal incompetency with regard to participation in this study	
No informed consent	
Additional exclusion criteria for subjects undergoing MRI‐scans	
Incompatible implants in the body (such as cochlear implants, insulin pumps, a copper IUD, or other metal implants)	
Any risk of having metal particles in the eye, due to manual work without proper eye protections	
Claustrophobia	
The refusal to be informed of structural brain abnormalities that could be detected during the experiment	
No informed consent	

Abbreviatons: BSSI: Beck Scale for Suicide Ideation, CYP: cytochrome P450, GHB: gamma‐hydroxybutyric acid, IUD: intrauterine device, MAO‐I: monoamine oxidase inhibitor, MRI: magnetic resonance imaging, PCP: phencyclidine.

As the aim of this study is to treat acute suicidality as a condition regardless of the presence of a DSM‐5 diagnosis, subjects with a range of psychiatric diagnosis (or without) are allowed to participate. However, subjects with a diagnosis of schizophrenia, or a history of other primary psychotic disorders are excluded. Although these patients may experience suicidal ideation, the psychotomimetic effects of ketamine may increase the chances of psychotic relapse. Subjects suffering from a depression with psychotic symptoms will not be excluded as a recent review has shown the risk of psychotic exacerbation to be limited in this diagnostic category (Veraart et al. 2021). Similarly, patients with bipolar I disorder are not excluded, as ketamine has not been shown to induce manic episodes in depressed bipolar patients relative to placebo (Diazgranados et al. 2010; Zarate et al. 2012).

Patients with a history of ketamine‐ or phencyclidine (PCP) addiction will be excluded. Importantly, patients with histories of substance abuse, other than ketamine or PCP, will not be excluded from the study. This decision is based on the recognition that individuals who experience suicidal thoughts or attempt suicide often have co‐occurring substance abuse disorder and are often under the influence of a substance when performing a suicide attempt. By excluding these patients, who may profit from ketamine, we would limit ketamine's potential benefit. In addition, recent studies have shown beneficial results of ketamine also as a treatment for substance abuse (Goldfine et al. 2023; Dakwar et al. 2019, 2014). However, there may also be a risk of developing a ketamine addiction, which will be carefully monitored.

### 2.4 Procedure

**FIGURE 1:**
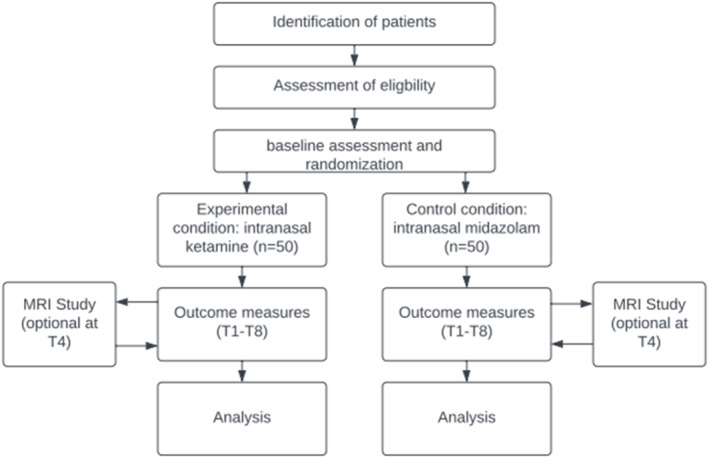
Trial Flowchart. T numbers are explained in Table 2.

For a schematic overview of the study procedures, see Figure 1, which represents the flowchart of the study procedures and Table 2, which shows at which time points study procedures are performed. When a suicidal patient is evaluated at the psychiatric emergency ward, or when an already admitted patient becomes suicidal, or when a patient visiting the outpatient clinic of the UMCG or Lentis presents with acute suicidality, the patient will be seen per care as usual. The treating physician will perform standard care and a preliminary screening. Standard care for acute suicidality consists of psychiatric assessment, often followed by a supportive/psychotherapeutic intervention, in which family members or close friends are involved. Sometimes sedating medication is administered as complementary treatment.

**TABLE 2:** (Study procedures).

Study procedure	Outcome	Study period^a^											
		Enrollment	Allocation	Post allocation									Close out
		Screening	Baseline	T0	T1	T2	T3	T4	T5	T6	T7	T8	Tx
Screening/informed consent	—	**x**											
Administration of study medication.	—			**x**									
End of follow‐up	—												**x**
**Measuring instrument** ^b^:	—												
BSSI	Suicidal ideation	**x**	**x**			**x**	**x**	**x**	**x**	**x**	**x**	**x**	
MADRS	Depression symptoms	**x**	**x**			**x**	**x**	**x**	**x**	**x**	**x**	**x**	
CGI	Overall disease severity and change		**x**			**x**	**x**	**x**	**x**	**x**	**x**	**x**	
RASS	Sedation/agitation						**x**						
CTQ‐SF	Childhood trauma							**x**					
SAFTEE	Side effects		**x**			**x**	**x**						
MINI	Establishing DSM‐5 diagnoses							**x**					
Physical examination	Blood pressure, O2 saturation, heart rate, temperature		**x**		**x**	**x**							
Blood collection	Biomarkers, gene expression, ketamine levels		**x**			**x**							
MRI/fMRI/MRS	Structural MRI, functional MRI (fMRI), diffusion tensor imaging (DTI), ^1^H‐MRS‐analysis of glutamate in hippocampus and prefrontal cortex							**x**					
Demographic questionnaire	Demographics		**x**										
Anamnesis/dossier study	Suicide attempts, successful suicides, admission duration					**x**	**x**	**x**	**x**	**x**	**x**	**x**	

^a^ The time points correspond with the following real time moments: Screening (SC), baseline (BL), moment last nasal spray is given (T0) and 30 min (T1) 60 min (T2), 180 min (T3), 1 day (T4), 3 days (T5), 1 week (T6), 2 weeks (T7) and 4 weeks (T8) after study medication is given and close out (Tx).

^b^ Beck scale for suicidal ideation (BSSI), Montgomery Åsberg Depression Rating Scale (MADRS), Clinical Global Impression (CGI), Richmond Agitation and Sedation Scale (RASS), Childhood Trauma Questionnaire Short Form (CTQ‐SF), Systematic Assessment for Treatment Emergent Effects (SAFTEE), Mini‐International Neuropsychiatric Interview (MINI), Magnetic Resonance Imaging (MRI), Functional Magnetic Resonance imaging (fMRI), Magnetic Resonance Spectroscopy (MRS).

Following the initial screening by the treating clinician, potentially eligible participants are informed about the trial. If a patient is interested to receive more detailed information about the study, a pre‐assessment by a member of the research team is performed to investigate if the subject meets the inclusion‐ and exclusion criteria (Table 1) and wants to participate. Given the acute nature of the study, a subject has at minimum 1 hour to decide whether he or she wants to participate (if an inclusion spot is available the same day and the patient is determined to participate) but in most cases the patient will be given one or more days to consider participation. In that case, a call will be planned to confirm participation and the inclusion date will be planned. Then, informed consent will be obtained and a treatment date will be planned. Patients are allowed to resign from the study at any moment without providing a reason. When medical situations or the patient's circumstances for other reasons require study withdrawal, the study clinicians will pursue such action. Subjects will not be replaced after withdrawal.

Following inclusion, a general physical examination and an evaluation of mental competence, as assessed by the Koninklijke Nederlandse Maatschappij tot bevordering der Geneeskunst (Royal Dutch Medical Association) (KNMG) directive, will be performed (Wilsbekwaamheid | KNMG). An assessment of psychiatric and somatic condition, body weight, temperature, blood pressure, pulse, alcohol consumption (a respiratory alcohol test, in case the subject reports to have drunk) and pregnancy (urinary pregnancy test exclusively for women in reproductive age) will be performed. Furthermore, a urinary drugs test, in which presence of cocaine, amphetamines, ecstasy and cannabis will be assessed, will be administered. Of note, this urinary drug test will, given the time the analysis will cost, not be part of the screening. A baseline measurement with BSSI, which screens for eligibility, MADRS (Montgomery and Åsberg 1979), CGI (Guy 1976) SAFTEE (Levine and Schooler 1986) will be performed as primary and secondary outcome measures.

The subjects will then be admitted to the clinical psychiatry ward of the UMCG where study treatment will be given the same day. The subjects will remain hospitalized for 8–24 h after either IN ketamine or midazolam administration. As ketamine may have a profound subjective impact, it is considered important to let the subjects be hospitalized until the next day. Because of the sedating effect that both ketamine and midazolam can have, the subjects are asked to be accompanied by a relative or carer when they leave the hospital the next day. In case there is a reason for further hospitalization, this may be done as part of treatment as usual unrelated to the study protocol. To ensure attendance by nursing personnel, the decision was made to treat all patients on a closed ward. A disadvantage of this approach is that the setting of a closed ward may be perceived negatively by subjects and may thereby be a confounder, therefore this matter is discussed explicitly with potential subjects beforehand.

After a subject has arrived at the ward, he or she will be randomized to receive either ketamine or midazolam. Directly after the study drug has been administered, the subject may feel to be under influence. To ensure optimal support, the subject is asked whether he or she prefers the researcher to be present in the room, or on call in the hallway in front of the room. Before and after 60 and 180 min, 1 day, 3 days, 1, 2 and 4 weeks following the administration of the study drug, suicidality will be measured with the BSSI and the MADRS suicidality item. Also, the occurrence of suicide and suicide attempts, depressive symptoms as measured with the MADRS, clinical severity as measured by the Clinical Global Impression scale (CGI) will be assessed at these time points. Psychotomimetic and somatic symptoms will be measured with the SAFTEE at 60 and 180 min after study drug administration. The DSM‐5 diagnosis will be assessed with the Mini Neuropsychiatric Interview (MINI) and childhood trauma will be evaluated with the Childhood Trauma Questionnaire Short Form (CTQ‐SF) at one day after administration of study drug. Blood samples will be drawn at baseline (31 mL) and 180 min following administration of the study drug (22 mL) for the assessment of serum biomarkers of antisuicidal efficacy. A structural MRI, a functional MRI, diffusion tensor imaging (DTI), and quantitative proton magnetic resonance imaging (^1^H‐MRS) will be performed at 1 day after study drug administration. For a detailed overview see Table 2.

When the measurements of day 1 have been performed, after an evaluation of the treating psychiatrist of the ward, the patient is allowed to leave the hospital together with someone who is part of the subject's network.

### 2.5 Study Management

The study was approved by the Medical Ethical Committee of the University Medical Center Groningen. The trial is registered in the EU Clinical Trials Registry EudraCT under 2020‐002905‐24. The Clinical Research Office (CRO) of the UMCG and an independent Data Safety Monitoring Board (DSMB) will oversee the conduct of the study. The trial system will be audited two times per year by the CRO. The DSMB meets before the start of the inclusion, and after 25, 50, 75 and 100 inclusions.

### 2.6 Treatment

Treatment comprises the IN administration of either racemic ketamine (75 mg) or midazolam (4 mg) as an active placebo. Both treatments are administered in two nasal sprays (one for each nostril (ketamine: 12.5 mg; midazolam 0.67 mg) at three times, totalling 75 mg of ketamine and 4 mg of midazolam. In line with previous studies (Lapidus et al. 2014; Gálvez et al. 2018), the three sets of two nasal sprays are administered 10 min apart each to optimize ketamine resorption. IN administration was selected because it is considered a more practical route of administration for both the patient and the medical professional, because the onset is fast, and administration is relatively easy (Meerwijk et al. 2016). The decision was made to include midazolam as an active placebo to ensure better blinding conditions. Hence, the sedating effects mimic those of ketamine, yet no antidepressant, nor antisuicidal effect has been established (Boutron et al. 2006; Murrough et al. 2013).

The selected dosage of racemic ketamine was derived from the relatively lower bioavailability of IN ketamine (45%) (Yanagihara et al. 2003) relative to intravenous ketamine (100%). To reach the same plasma levels obtained with the standard 0.5 mg/kg IV administration, a dose of 1.03 mg/kg which is about 72 mg in a 70 kg person, should be administered. Consequently, an intranasal dosage of 75 mg would, on average, yield similar ketamine plasma levels as the commonly used 0.5 mg/kg dosage for IV ketamine in the treatment of depression. Moreover, in intravenous studies no serious adverse events have been reported. The adverse effects that were reported were minor dissociative symptoms and small changes in systolic blood pressure. Therefore, a dose of 75 mg IN ketamine is considered safe and potentially effective (Wan et al. 2015; Patil and Anitescu 2012).

## 3 Objectives

### 3.1 Primary Objective

To test the hypothesis that a dose of 75 mg of intranasal ketamine lowers suicidal ideation and behavior significantly more than active placebo: midazolam.

### 3.2 Secondary Objectives

To obtain (1) an understanding of correlates of the potential effect of ketamine, and (2) an understanding of the underlying mechanisms via which ketamine might exert its antisuicidal effect.

### 3.3 Outcome Parameters

#### 3.3.1 Primary Outcome Parameter

The primary outcome parameter comprises the score of the Beck Scale for Suicidal Ideation (BSSI) at 180 min in the ketamine relative to the midazolam (active placebo) group.

#### 3.3.2 Secondary Study Parameters

The secondary study parameters include a comparison of the ketamine relative to the midazolam group on: 1. Suicidality (BSSI, MADRS item 10), 2. Suicides and suicidal acts (*n*, %), 3. Admission (n), 4. Depressive symptoms (MADRS), 5. Clinical impression (CGI), 6. Psychotomimetic and somatic symptoms (SAFTEE), 7. DSM‐5 diagnosis (MINI), 8. Childhood trauma (CTQ), 9. Blood‐based biomarkers, 10. Plasma ketamine concentrations, 11. Neuroimaging markers, 12. Responder/non responder (*n*, %), 13. Correlation between BSSI and MADRS scores, 14. Association sex and changes in BSSI scores.

#### 3.3.3 Serious Adverse Events (SAEs)

A serious adverse event is any untoward medical occurrence or effect that:

– results in death;
– is life threatening (at the time of the event);
– requires hospitalization or prolongation of existing inpatients' hospitalization;
– results in persistent or significant disability or incapacity;
– is a congenital anomaly or birth defect; or any other important medical event that did not result in any of the outcomes listed above due to medical or surgical intervention but could have been based upon appropriate judgement by the investigator.

An elective hospital admission will not be considered as a SAE.

The sponsor will report the SAEs through the web portal *ToetsingOnline* to the accredited Institutional Review Board (IRB) that approved the protocol, within 7 days of first knowledge for SAEs that result in death or are life threatening followed by a period of maximum of 8 days to complete the initial preliminary report. All other SAEs will be reported within a period of maximum 15 days after the sponsor has first knowledge of the serious adverse events.

The investigator will report all SAEs without undue delay after obtaining knowledge of the events, except for the following SAEs: SAEs—with the exception of mortality—that take place more than 20 days after the ketamine/midazolam nasal spray administration, since SAEs that occur more than 20 days after administration of the nasal spray are unlikely to be related to the contents of the nasal spray. In a 2015 Cochrane review, no evidence was found for any efficacy or side effect of ketamine at points of time more than 14 days after ketamine administration (Caddy et al. 2015). Given its elimination half‐life of 2.3 h, midazolam is unlikely to cause any effects more than 20 days after administration (Heizmann et al. 1983). These non‐fatal SAEs that take place more than 20 days after the intervention will be reported in an overview list (line listing) every half year to the IRB. Hence, SAEs in terms of mortality will be reported throughout the study.

However, ketamine abuse could occur later than 14 days after administration of ketamine as described in a case report by Bonnet (Bonnet 2015). Because of this, these events will be marked as (serious) adverse events based on the individual case.

#### 3.3.4 Suspected Unexpected Serious Adverse Reactions (SUSARs)

Adverse reactions are all untoward and unintended responses to an investigational product related to any dose administered.

Unexpected adverse reactions are SUSARs if the following three conditions are met.

1. The event must be serious (see chapter 9.2.2);
2. There must be a certain degree of probability that the event is a harmful and an undesirable reaction to the medicinal product under investigation, regardless of the administered dose;
3. The adverse reaction must be unexpected, that is to say, the nature and severity of the adverse reaction are not in agreement with the product information as recorded in:
  – Summary of Product Characteristics (SPC) for an authorized medicinal product;
  – Investigator's Brochure for an unauthorized medicinal product.

The sponsor will report expedited the following SUSARs through the clinical trial web portal *ToetsingOnline* to the IRB:

– SUSARs that have arisen in the clinical trial that was assessed by the IRB;
– SUSARs that have arisen in other clinical trials of the same sponsor and with the same medicinal product, and that could have consequences for the safety of the subjects involved in the clinical trial that was assessed by the IRB.

The remaining SUSARs are recorded in an overview list (line‐listing) that will be submitted once every half year to the IRB. This line‐listing provides an overview of all SUSARs from the study medicine, accompanied by a brief report highlighting the main points of concern.

The expedited reporting of SUSARs through the web portal *Eudravigilance* or *ToetsingOnline* is sufficient as notification to the competent authority.

The sponsor will report expedited all SUSARs to the competent authorities in other Member States, according to the requirements of the Member States.

The expedited reporting will occur not later than 15 days after the sponsor has first knowledge of the adverse reactions. For fatal or life‐threatening cases the term will be maximal 7 days for a preliminary report with another 8 days for completion of the report.

### 3.4 Measurements

#### 3.4.1 Questionnaires

##### 3.4.1.1 Suicidality

The BSSI is used for assessing suicidality (Beck et al. 1979). The BSSI comprises a frequently used measure of suicidality in research on the antisuicidal effects of ketamine (Beck et al. 1979; Abbar et al. 2022; Price et al. 2014). The BSSI is a 21‐item questionnaire in which each item is given a score of either 0, 1 or 2, with a maximum score of 42 which can be either administered by the clinician or by the patient himself. In our study we use the clinician administered version. If the subject scores > 0 on items four or 5, the questionnaire proceeds with items 6‐19 and afterwards items 20 and 21. If item 4 and 5 are scored 0, the questionnaire immediately proceeds to items 20 and 21. The BSSI comprises items about the wish to live (items 1, 3 and 5), the wish to die (items 2 and 4), the duration and frequency of the thoughts (items 6 and 7), the acceptance of suicide (item 8), self‐control in regard to suicide (item 9), fear of consequences of suicidality (item 10), the reason for suicidality (item 11), preparation for suicidality (items 12, 13, 16, 17 and 18), the courage to commit suicide (item 14), the expectancy in regard to committing suicide (item 15), the sharing of suicidal thoughts (item 19) and the history of suicide attempts (items 20 and 21).

The BSSI has been validated across several domains (Beck et al. 1979). The scale's scores correlated well with other suicide scales or measures. The BSSI also discriminated well between patients with a depression with and without suicidality. Construct validity was studied by evaluating correlations between depression scores on the Beck Depression Inventory (BDI) and BSSI scores and scores on the hopelessness scales (HS) and the BSSI. Sensitivity over time was tested by correlating BSSI scores prior to outpatient treatment with scores after treatment. These scores showed a significant Spearman's rho (0.51, *p* < 0.05). Furthermore, a factor analysis was performed from which three factors emanated to which the individual items pertained: Factor I: Active Suicidal Desire, Factor II: Preparation, Factor III: Passive suicidal desire. Later, a self‐administered version of the BSSI was developed as well, which was valid in regard to the clinician administered version (Beck et al. 1988).

For the present study the original English version was translated to Dutch and linguistically validated by back translation. The suicidality item from the MADRS (item 10) was used as a complementary measure for suicidality.

##### 3.4.1.2 Depressive Symptoms

The MADRS is used for assessing depressive symptoms (Montgomery 1979). The MADRS is a 10‐item instrument which is filled out by the clinician or researcher which was designed to detect treatment effects since the rating scales up to that date mainly reflected diagnostic features of depression which were not particularly sensitive to change. The scale was constructed based on the Comprehensive Psychopathological Rating Scale (CPRS) (Åsberg et al. 1978), which comprises 65 items. Of these items, the 17 most occurring in a Swedish sample were selected. Then, of these 17 the 10 most sensitive items were selected. The items can be scored from 0 through 6, with a maximum total score of 60.

The validity was tested by assessing inter‐rater reliability and by determination of the correlation with the Hamilton Depression Rating Scale (HDRS) (Montgomery 1979). The inter‐rater reliability is adequate. This was tested by comparing ratings of two English raters, two Swedish raters and the ratings of an English and a Swedish rater. In addition, experienced raters were compared to non‐experienced raters, which also showed high reliability. Also, the MADRS was studied for validity, and showed significant correlations with the HDRS and the global judgement of clinicians.

### 3.5 Clinical Impression

The CGI (Guy 1976) is a metric based on the impression the clinician or researcher has about severity and the improvement of the disease. No information about validation could be retrieved. However, Ruhé et al. (2005) found the CGI to be less reliable than the Maier Bech and Hamilton Depression Scales (Ruhé et al. 2005). However, the authors note that the reverse would be true if the CGI was used as the gold standard.

### 3.6 Psychotomimetic and Somatic Symptoms

Psychotomimetic and somatic symptoms are measured with the SAFTEE (Levine and Schooler 1986), a 57‐item instrument that systematically studies psychotomimetic and somatic symptoms comprising items about, the head, eyes, ears, mouth, nose and throat, chest, skin and cardiovascular, gastrointestinal, genitourinary and musculoskeletal, psychological and ‘other’ items. This questionnaire was developed as a broadly accepted questionnaire which systematically evaluated side effects of pharmacological treatments. Both the general inquiry (GI) and the specific inquiry version of the SAFTEE showed good interrater reliability (kappa scores of 0.95 and 0.88 respectively) (Jacobson et al. 1986). In yet another study, nurses were shown to be in agreement in regard to ratings on the SAFTEE general inquiry and SAFTEE scores were equivalent to scores on the Association for Methodology and Documentation in Psychiatry Somatic Signs (AMDP‐SS) questionnaire (Guy et al. 1986).

### 3.7 Childhood Trauma

Childhood trauma will be assessed with the Childhood Trauma Questionnaire Short Form (CTQ‐SF) (Bernstein et al. 2003). This is a validated questionnaire that consists of 25 items that can be rated from 1 to 5, with a maximum score of 125. These items assess 5 dimensions of childhood maltreatment: (1) Physical Abuse, (2) Emotional Abuse, (3) Sexual Abuse, (4) Physical Neglect, (5) Emotional Neglect. The Dutch version was validated relative to the original version (Thombs et al. 2009). The Chronbach's alpha for the Dutch and the English CTQ‐SF's were 0.91 for Physical Abuse, 0.89 for Emotional Abuse, 0.95 for Sexual Abuse, 0.63 for Physical Neglect and 0.91 for Emotional Neglect.

### 3.8 Laboratory Assessment

Blood samples are collected at baseline and 180 min following administration. On each time point three tubes are collected for assessment of the levels of relevant biomarkers in serum (1 × 10 mL serum‐separator tube), ketamine concentrations in plasma (2 × 6 mL EDTA) (Heizmann et al. 1983). An additional blood sample will be collected for genotyping of polymorphisms relevant to ketamine metabolism and efficacy (1 × 9 mL EDTA). Following pre‐processing, the samples will be stored at −80 degrees Celsius until the laboratory assessment is performed using an enzyme‐linked immunosorbent assay (ELISA). After the laboratory tests have been performed, the remaining blood will be stored in the biobank of the UMCG. See supplement for details.

### 3.9 Neuroimaging

Magnetic resonance imaging (MRI) will be performed to assess hippocampal volumes. MR‐based spectroscopy (MRS) will be additionally performed to evaluate glutamate levels in the hippocampus and pre‐frontal cortex. To further test whether these structural assessments influence the integration of the hippocampus within the fronto‐limbic circuitry that is thought to underlie suicidality, structural connectivity using diffusion tensor imaging (DTI) and resting‐state functional connectivity will be measured using functional magnetic resonance imaging (fMRI) ((Valentine et al. 2011; Li et al. 2020; Kraguljac et al. 2017)).

### 3.10 Statistical Analyses

Statistical analyses will be based on the intention‐to‐treat principle. Baseline characteristics of the ketamine and midazolam condition will be compared using independent samples *t*‐tests and chi‐square tests for continuous and categorical variables respectively. Association patterns between continuous variables will be expressed in Pearson's correlation coefficients or Spearman's correlation coefficients, where appropriate (Lee Rodgers and Alan Nice Wander 1988; Spearman 1904). The primary analysis consists of a comparison between the treatment groups of the primary clinical outcome (BSSI) 180 min after the IN ketamine or active placebo administration. An analysis of covariance (ANCOVA) will be performed to evaluate the influence of treatment (ketamine vs. midazolam) as independent variable on the BSSI score 180 min following administration as the dependent variable while controlling for baseline BSSI score as covariate (Guy et al. 1986). Additionally, the scores at baseline and following treatment will be further investigated using additional ANCOVA analyses that includes BSSI baseline values and treatment center as covariates (Vickers and Altman 2001).

Regarding the secondary clinical outcomes, we will compare the change in the BSSI, MADRS, the CGI and SAFTEE from baseline to all timepoints between the ketamine and midazolam condition using linear mixed model analyses. The number of suicides during the study period in both treatment arms will be analyzed using the Chi‐square (Pearson 1900) or Fisher's exact test (Fisher 1934) where appropriate. In all analyses statistical uncertainty will be expressed in 95% confidence intervals (CI). We will perform a responder/non‐responder analysis with regard to baseline characteristics. To assess the relation between suicidality and depressive symptoms, the change in BSSI scores will be correlated with the changes in MADRS scores and sex will be correlated with change in BSSI.

A repeated measures analysis of variance (ANOVA) will be performed to investigate the change in serum biomarker levels from baseline to 180 min following administration. Pearson correlation analyses will further be performed to assess the association between the biomarker levels and BSSI scores. The correlation between the plasma ketamine concentration and change in BSSI score will be equally evaluated using a Pearson correlation analysis.

Results concerning the neuroimaging data will be acquired using appropriate analysis methods for each MRI modality: resting‐state will be analysed using independent component analysis (ICA), fractional anisotropy (FA) analysis to analyse DTI and LCModel to analyse ^1^H‐MRS‐spectra (Provencher 1993). Independent samples *t*‐tests will be used to compare differences in neuroimaging data between ketamine responders and non‐responders. In addition, correlations will be analyzed between neuroimaging data and BSSI scores (after ketamine) to explore neuroimaging markers of suicidality.

### 3.11 Missing Data

Missing data will be handled using complete case analysis, where only cases with no missing values for the variables of interest will be included in the analysis.

### 3.12 Sample Size Calculation

Previous studies evaluating the antisuicidal effect of ketamine report a medium‐to‐large effect size (*d* = 0.60–0.82) (Price et al. 2009; Grunebaum et al. 2018; Canuso et al. 2018). The presented clinical trial includes patients that are suicidal without necessarily meeting the DSM‐5 criteria for depressive disorders, and substance abuse is allowed to a certain extent. It is therefore expected that the effect size might be on the lower end of the described spectrum reported in previous studies. To ensure statistical power a more conservative, medium effect size was therefore considered (*d* = 0.60). Standardized methods for sample size calculation (G*Power 3.1) demonstrated a sample size of 45 patients per treatment arm for 80% power to detect a medium effect (*f* = 0.30), with a significance level of 5% (*α* = 0.05) when assuming a large correlation among two repeated measurements (*r* = 0.5) and no violation of the sphericity assumption (*ε* = 1) (Faul et al. 2007) Of note, the proposed sample size will be sufficient to correct for baseline BSSI score, since recent insights suggest that power increases rather than decreases as a result of correcting for baseline values (Holmberg and Andersen 2022). Anticipating on a 10% attrition rate, we will include (45/0.90 = ) 50 subjects per group (100 persons in total).

The selected effect size was based on several studies. Price et al. (2014) investigated the effect of ketamine on suicidal ideation in depressed patients in an RCT comparing subanaesthetic intravenously administered ketamine with intravenously administered midazolam as an active placebo (Price et al. 2014). After statistical adjustment, the researchers observed a substantial effect size of *d* = 0.82. Grunebaum et al. found an effect size of *d* = 0.75 with regards to reduction in suicidality scores at one day after administration (Grunebaum et al. 2018) and Canuso et al. (2018) found an effect size of *d* = 0.6 with regards to reduction of suicidality scores in a sample of depressed patients, at both 4 and 24 h after ketamine/placebo administration (Canuso et al. 2018).

For MRI outcome parameters a sample size is usually not determined by power analyses, but by prior experiences with similar protocols. Exact power calculations are difficult because of the complex “mass univariate” nature of brain imaging data as well as the difficulty to estimate effect sizes in different regions of interest beforehand. Furthermore, Thirion et al. (2007) used statistical models based on real and simulated data and concluded that in general, including 20 subjects per group is sufficient to obtain acceptable reliability in a group comparison fMRI study (Thirion et al. 2007).

### 3.13 Data Monitoring

An independent DSMB has been appointed, (see supplement for the DSMB‐charter). Given the vulnerability of the sample population and severity of the outcomes, interim data monitoring will be carried out at several instances during the trial. Analyses will be performed that can detect harmfulness of the treatment or evidence that the treatment has no tangible beneficial effect relative to the control condition.

Following recommendations by Freidlin et al. (2010), we will have a first interim monitoring moment once 25% of data is collected (t1: ‘harm look’). Here, inefficacy monitoring cannot be carried out given the size of the sample, but it will be evaluated whether the intervention leads to a significantly worse outcome than the control (Freidlin et al. 2010).

At 50% (t2) and 75% (t3) of data completion, inefficacy monitoring will be carried out, with the decision boundary being that the 95% CI of the treatment effect does not overlap with the effect that was expected in the trial design under the alternative hypothesis (i.e., intervention works better than control with a Cohen's *d* of 0.6). The general procedures described by Freidlin et al. (2010) will be used to guard against over‐aggressive testing (Freidlin et al. 2010).

### 3.14 Feasibility Pilot Study

Because this study involves a significant risk of both suicide and suicide attempts, and inferring that the procedure might be demanding, we conducted an open label pilot feasibility study in 12 patients. This pilot study revealed some issues that needed adaptation. The major issue observed within the original protocol arose from the necessity to include a patient within 24 h of the exacerbation of suicidality, which meant that the subjects had to be included on the same day as on which they were identified. Also, severely suicidal patients who had been even more suicidal more than one day before the inclusion, had to be excluded. As a result of this, potential inclusions were missed who in our view fitted the goals of this study and represented clinical reality. Therefore, we decided to adjust the period before inclusion in which suicidality should have increased to 72 h (and 96 h before the administration of the ketamine/midazolam). Also, we decided to leave out blood pressure, pulse, temperature and saturation measures after T1 hours, since in the pilot study we noticed no notable differences at these time points from baseline. Furthermore, after advice of the DSMB we decided to extend follow up with the time points 2 and 4 weeks in order to better monitor potential ketamine abuse after study participation. Finally, we decided to leave the Clinician Administered Dissociative States Scale (CADSS) out, since we considered it redundant. The results of the pilot will be published in detail elsewhere.

## 4 Discussion

The aim of the clinical trial described in the present study protocol is to determine whether one IN treatment of ketamine significantly decreases suicidality relative to midazolam as an active placebo in acutely suicidal subjects. The study protocol was designed to not only evaluate this primary research question, but also to explore potential mechanisms underlying the proposed antisuicidal effects of ketamine. The procedures were refined based on an initial pilot study. Several remarks can be made regarding the design of studies on acute suicidality as a transdiagnostic condition and, hence, the future interpretation of results.

The central research question in this study is whether ketamine improves suicidality as a phenomenological entity irrespective of the specific concurrent psychiatric diagnosis. In the last two decades, numerous studies have evaluated ketamine as an antidepressant and based on the collated evidence, it can be concluded that ketamine indeed yields a fast and thorough antidepressant effect (McIntyre et al. 2020). In these studies in depressed subjects, a profound antisuicidal effect was also demonstrated (Wilkinson et al. 2018). However, in most previous studies patients with acute suicidality have been excluded resulting in a lack of knowledge about this specific patient group (aan het Rot et al. 2010; Price et al. 2009; Lapidus et al. 2014; Singh et al. 2016). While initial studies focussing on acute suicidality have been published (Domany and McCullumsmith 2022; Abbar et al. 2022) it remains unclear which subgroups may benefit most from ketamine treatment and which administration route is most effective and practical. Some studies evaluating ketamine treatment in suicidal patients not suffering from major depressive disorder (MDD) have been performed (Abbar et al. 2022; Domany and McCullumsmith 2022) but these studies were performed with saline as a placebo and were either performed with IV ketamine or had a relatively small sample size. Therefore, several important knowledge gaps still exist. We thus believe it is critical to perform a larger, well‐powered double‐blind active placebo‐controlled study in a real‐world setting to determine whether ketamine indeed has an antisuicidal effect regardless of a specific underlying diagnosis. If that were the case, ketamine could be prescribed for this group, with the aim to shorten suffering and prevent actual suicide.

Studying the general antisuicidal effects of ketamine may further deepen our understanding of suicidality as a separate phenomenon. Important aspects of suicidality may be missed by only considering it as a symptom of other disorders (e.g., depression, personality disorder, schizophrenia). Notwithstanding, approaching suicidality as a separate diagnostic entity constitutes its own difficulties. To agglomerate different forms of suicidality might also be counterproductive, since different kinds of suicidality (e.g., emerging from depression or from personality disorder) may have different aetiologies that could require different clinical strategies. In relation, it remains uncertain whether suicidality can indeed be treated successfully with ketamine transdiagnostically. Nonetheless, should a disparate response to ketamine from different (DSM‐5)‐diagnostic categories emerge in this study, we believe that this could contribute to our understanding of suicidality, and as such could be a valuable result of this study.

In order to mimic the natural, practical setting of psychiatric emergency units, we decided to administer a racemic ketamine nasal spray instead of the more widely studied ketamine infusion. The IV‐route offers the highest (100%) and most reliable bioavailability (Clements et al. 1982), but poses practical challenges. Interestingly, Abbar et al. (2022) studied IV racemic ketamine for acute suicidality (Abbar et al. 2022) and found promising results. Our study will be different in that subjects will be admitted much shorter (one night) than in this IV study in which subjects were admitted for longer periods. Furthermore, in the IV study subjects received two ketamine/placebo infusions, whereas we will administer the IN ketamine/placebo in one session. Importantly, a recent review concluded that IN esketamine may not yield an antisuicidal effect as opposed to IV racemic ketamine (Jollant et al. 2023). One of the reviewed studies showed that IN racemic ketamine when compared to placebo led to a significant reduction of the MADRS suicide item, and a non‐significant reduction on the BSSI compared to placebo (Domany and McCullumsmith 2022). Given the outcome of this study and given the fact that we also use racemic IN ketamine instead of IN esketamine, we believe our study will still be valuable contribution to the literature. Another alternative could be to use oral ketamine in our study population (Schoevers et al. 2016; Smith‐Apeldoorn et al. 2022, 2024), but this has a somewhat longer interval to reach maximal concentration (*T*_max_) and has lower bioavailability. Hence, IN ketamine is absorbed rapidly and can bypass the blood brain barrier (Andrade 2015). On the other hand, a potential disadvantage of the early *T*_max_ may be a higher abuse potential, and a bad experience may be harder to forestall in patients that have higher intranasal bioavailability for ketamine, depending on the permeability of their nasal mucosa. Furthermore, providing subjects with a fixed dose (75 mg) instead of a more precisely titrated dose of 0.5 mg/kg as seen in IV trials, may pose another relative disadvantage although this also facilitates a rapid administration. Finally, saline was used as a placebo, whilst we use midazolam as an active placebo, since this improves the reliability of the outcome, and will depict ketamine's antisuicidal effect more reliably.

A potential problematic aspect of clinically applied ketamine is its potential for addiction which can have devastating consequences when abused in high doses and high frequency (Liu et al. 2016; Strous et al. 2022). Therefore, additional care should be in place regarding the potential emergence of ketamine addiction as a result of clinical ketamine use. In the current study design, we have taken the following precautions. First, patients with a known history of ketamine or PCP addiction will be excluded. Second, patients are informed about the risk of developing ketamine addiction before study participation, and they are informed to report craving for ketamine or considering illegally acquiring ketamine so that professional help can be arranged. Lastly, during the 1 month of follow‐up our study team will specifically ask for (signs of) ketamine abuse. We decided not to exclude other forms of substance abuse such as a history of alcohol use disorder because this is a common comorbidity in depression and other mental disorders as mentioned above. We are currently unaware of data showing that people with a history of addiction are at an increased risk to develop an addiction for ketamine as well. Although this seems plausible, ketamine has been evaluated as a treatment for addictions of several substances, with studies showing promising results (Goldfine et al. 2023; Dakwar et al. 2019, 2014). Notwithstanding, in some patients with a history of addiction a risk may exist for developing a ketamine addiction. Given the grave nature of suicidality and suicide, we consider this risk to be acceptable when monitored as described above. Many medical treatments would involve some form of risk, which is weighed against the potential positive effects and also depends on whether adequate follow‐up measures are taken.

Since we will obtain data on blood‐based and imaging biomarkers, our study may additionally provide knowledge about neurobiological aspects of suicidality: suicidality independent from the underlying diagnosis may respond comparably to ketamine as suicidality in MDD, or it may not respond at all. Recently, a meta‐analysis about blood‐based biomarkers in MDD has been published (Medeiros et al. 2022). In this meta‐analysis 460 blood‐based biomarkers comprising neurotrophic and inflammatory markers, ketamine levels and levels of ketamine metabolites, markers of the tryptophan kynurenine pathway, genetic markers, amino acids genetic markers, genetic expression patterns and metabolomic patterns were studied. Only BDNF showed a statistically significant longitudinal increase following ketamine administration in MDD patients who responded to treatment. Although previous evidence suggested that ketamine could have anti‐inflammatory effects (Zanos and Gould 2018; Zanos et al. 2018), this meta‐analysis failed to find evidence for this theory. The same group published a systematic review about brain‐based correlates of an antidepressant response to ketamine (Medeiros et al. 2023). In three independent samples they found post‐treatment gamma power increases in frontoparietal regions, post‐treatment increases in functional connectivity within the prefrontal cortex and a post‐treatment increase in striatum activation. Potential insights from our study, in which we will both study blood‐based biomarkers and neuroimaging markers, may add to the mechanistic understanding of the potential antisuicidal effect of ketamine which may aid its clinical management.

A challenge that we will encounter in the proposed trial is how to deal with suicidality that re‐emerges when the desired effect of ketamine wears off. The antidepressant effect of a single ketamine treatment typically lasts between 3 and 7 days (Kishimoto et al. 2016) and the severity of suicidal symptoms may then return to the pre‐treatment level. Because our study comprises only a single administration, subjects cannot be expected to remit for a substantial period. This short‐lived remission of suicidality should therefore be used as a window of opportunity to reach important treatment goals by means of psychosocial or psychotherapeutic interventions. Based on the results of our pilot study (submitted), we expect subjects to be more open to these interventions that would be provided by their care providers, than they would have been if they had not received ketamine. However, in case suicidality falls back to a level of before the administration of the study drug, or even to a more precarious level, an admission to a psychiatric ward or an extensive psychosocial intervention will be considered. The KETA‐study is an add‐on study, so all patients that are enrolled will also have a treating physician who will provide ongoing regular treatment. Additionally, these physicians will be made aware of the fact that subjects could be as suicidal or more suicidal after the study drug wears off, than they were before the drug was administered.

In conclusion, the authors anticipate that results from the presented RCT will provide further knowledge for the treatment of patients suffering from acute suicidality. Although there are substantial challenges, with regard to potential abuse, the disadvantages of IN dosing and the potential rebound suicidality, this clinical trial will provide valuable insights into practical application, clinical aspects, and mechanistic substrates of IN ketamine for patients suffering from acute suicidality.

## Author Contributions

**Jurriaan F. M. Strous:** conceptualization, data curation, investigation, methodology, project administration, formal analysis, funding acquisition, software, validation, visualization, writing – original draft, writing – review and editing, **Gijs H. J. Roelandt:** conceptualization, data curation, investigation, methodology, project administration, formal analysis, funding acquisition, software, validation, visualization, writing – review and editing, **Jens H. van Dalfsen:** formal analysis, methodology, supervision, writing – review and editing, **Jeanine Kamphuis:** supervision, writing – review and editing, **Radboud M. Marijnissen:** supervision, writing – review and editing, **Robert A. Schoevers:** conceptualization, funding acquisition, resources supervision, writing – review and editing.

## Ethics Statement

Before inception of the study all relevant documents have been submitted to the ethical review board of the University Medical Center Groningen (METC 2020/378), which concluded that the project is in line with the Dutch law (NL74304.042.20). The KETA‐study has been registered at the EU Clinical Trial Register (EudraCT 2020‐002905‐24). The study is conducted in accordance with the declaration of Helsinki (World Medical Association declaration of Helsinki 2013). Substantial amendments will be submitted to the Institutional Review Board of the UMCG. All researchers and recruitment sites will be notified.

## Consent

All patients receive both oral and written information about the study. This information comprises the scope and relevance of the study, as well as the patient burden and confidentiality issues. Participants have the possibility to ask research staff for clarification of questions regarding the study. Before participation, written informed consent is obtained at least one hour after patients have been informed in written and oral form.

## Conflicts of Interest

The authors declare no conflicts of interest.

## Supporting information


Supporting Information S1

Supporting Information S2

Supporting Information S3

Supporting Information S4

Supporting Information S5

Supporting Information S6

Supporting Information S7

Supporting Information S8

## Data Availability

The data that support the findings of this study are available on request from the corresponding author. The data are not publicly available due to privacy or ethical restrictions.
