# Supplementary material for: The Ketamine Trial for Acute Suicidality (KETA): Study Protocol of a Double‐Blind Randomized Placebo‐Controlled Superiority Trial on Intranasal Racemic Ketamine Compared to the Active Placebo Intranasal Midazolam as Treatment for Acute Suicidality
Source: Int J Methods Psychiatr Res. 2025 Nov 19;34(4):e70044. doi: 10.1002/mpr.70044 (PMC12627964; doi:10.1002/mpr.70044)
Supplement: Supplementary file 1 — Supporting Information S1 [file MPR-34-e70044-s004.docx]

**Supplement 1 - Charter Data and Safety Monitoring Board (DSMB)**

**Ketamine Trial for Acute suicidality (KETA)**

Page Break

| **Full title of the study: Efficacy and feasibility of intranasal ketamine on acute suicidality, a multicenter double blind randomized placebo-controlled trial (Ketamine Trial for Acute suicidality KETA)** |
| --- |
| **Short title of the study: (Ketamine Trial for Acute suicidality, KETA)** |
| **ABR number:**NL 74304 |
| **Research protocol**    Version number: 1.0  Date: 2-3-2022 |
| **Planned study period**    Start study: 1-10-2022  End study: 1-10- 2025 |
| **DSMB members**    Name DSMB clinician (Chair):  Daniëlle Cath  Medical specialty:                     Psychiatry  Contact data:      daniëlle.cath@ggzdrenthe.nl      Name DSMB pharmacist:           Eric van Roon  Medical specialty:                       Hospital pharmacist-clinical pharmacologist  Contact data:          e.n.van.roon@mcl.nl        Name DSMB statistician:          Frederike Jörg  Contact data:              f.jörg@umcg.nl |
| **Principal investigator**    Name:                                      Prof. dr. Robert Schoevers  Department:                             Psychiatry UMCG |

| **Objective of the study**    To assess whether a dose of 75mg intranasal ketamine reduces acute suicidality relative to a 4mg intranasal dose of the active placebo midazolam, 180 minutes after administration. |
| --- |
| **Type of study**    Monocenter  X Multicenter Number of sites, including UMCG:2    Name principal investigator participating center 1: Prof. dr. Robert Schoevers  Department, Hospital: Psychiatry, University Medical Center Groningen.    Site 2  Lentis Groningen  Name principal investigator participating center 2. Dr. Bennard Doornbos |
| **Planned number of participants (total and per center):**  100 (50) |
| **Planned period of observation**    Moment of inclusion of first patient included:*01-10-2022*  Moment of follow-up of last patient included:*01-10-2025* |
| **Risk of the study**       negligible risk  X moderate risk     high risk    **Motivation of risk assessment:**  Although we assess the risk of our intervention with regard to safety as negligible, we are aware that suicidal patients may commit suicide and that there is a significant chance that a suicide may occur. |

**1. Scope of the charter**

This charter will describe the primary responsibilities of the DSMB, its membership and the purpose and timing of the meetings. The charter will also provide the methods of providing information to and from the DSMB and the decision-making process of the DSMB. All amendments will be documented. Each revision will be agreed upon by the Principal Investigator and DSMB. In case of revision of the charter the METC will be notified.

1. **Aims of the DSMB**

The DSMB will act in an independent, expert and advisory capacity to monitor participant safety, and evaluate the overall conduct of this study.

1. **Responsibilities of the DSMB**

The DSMB members should be familiar with the protocol and other documents related to the study; they are obliged to review and approve the DSMB charter.

The specific responsibilities of the DSMB are to:

▪ Monitor safety data, specifically the number of suicides or suicidal acts, and other (serious) adverse events on a regular basis and, if required, on ad hoc basis to guide recommendation for continuation of the study or early termination because of clear harm.

▪ Monitor efficacy of the intervention after 25, 50, and 75 inclusions:

▪ Evaluate the overall conduct of the study,

o compliance with the protocol by participants and investigators

o recruitment figures

o reports on completeness of data

o the monitoring report(s)

3.1 Confidentiality

DSMB members will not share confidential information with anyone outside the DSMB, including the Principal Investigator. Strict confidentiality is also expected from the independent statistician (or statistical data analyst) who produces the unblinded interim report(s) for the DSMB. The DSMB members should store relevant documents safely after each meeting.

1. **DSMB membership**

The DSMB consists of 3 members: Daniëlle Cath, who is a psychiatrist and who will be the chair of the DSMB. Eric van Roon, a hospital pharmacist-clinical pharmacologist and Frederike Jörg, who is experienced in the methodological and statistical techniques for clinical research. All members are independent of the study.

DSMB membership is for the duration of the study. If a member leaves the DSMB during the course of the study the Principal Investigator and Chair will promptly appoint a new member with comparable expertise and qualifications as the DSMB member that is being replaced.

1. **Timing and purpose of meetings**

An initial meeting of the DSMB will be held prior to any participant enrollment in order for the DSMB members to fully understand the research protocol, to review and approve the DSMB charter, and to review the monitor plans for safety data.

A subsequent DSMB meeting will be held when one week follow-up is completed of 25 subjects ((1/4 of the sample), 50 subjects (1/2 of the sample) and 75 subjects (3/4 of the sample).

The following type of data per treatment group will be reviewed:

- relevant baseline data (age, sex, ethnicity, level of education, duration of psychiatric care, number of (failed) antidepressant treatments, number of earlier suicide attempts, DSM-V diagnosis).
- inclusion and exclusion criteria
- enrollment data, and protocol violations
- Safety data (specifically the number of serious adverse events in each treatment arm).

Besides the planned meeting the DSMB will perform ongoing safety surveillance. Any mortality or other serious adverse events will be directly reported to the DSMB and will be evaluated for possible relatedness to the study intervention.

5.1 Meeting format

DSMB meetings will be conducted face-to-face, consisting of an open and closed session.

*Open session:* This session will be attended by the Principal Investigator, representatives of the Study Group and – if needed - the independent statistician (or statistical data analyst). During this meeting the study team will provide general study information. The open session also provides the DSMB the opportunity to query the study team about issues that have arisen during their review of the data. Unblinded data will not be discussed in the open session.

*Closed session:* The closed session will include an assessment of safety data by treatment groups. Only DSMB members will be present.

*An ad hoc meeting* of the DSMB may be called at any time by the Principal Investigator or the DSMB if imminent subjects’ safety issues arise.

5.2 Meeting minutes

Meeting minutes will be kept for each meeting of the DSMB. The minutes of the open session will be prepared by the Principal Investigator and approved by the DSMB members. The minutes of the closed session will be prepared by the DSMB chair and approved by the DSMB members. The Principal Investigator and DSMB members should store the minutes safely.

1. **Preparation of interim report(s) to DSMB**

The interim report will consist of an Open Session Report and a Closed Session Report.

The *Open Session Report* will provide non-confidential, aggregated information in terms of baseline characteristics, inclusion and exclusion criteria, overall study progress (enrollment data and protocol violations) as well as number and type of (serious) adverse events. The open report will be prepared by the principal investigator or independent statistician (statistical data analyst) and will be distributed to the DSMB at least 2 weeks prior to the scheduled meeting.

*The Closed Session Report*will additionally provide the abovementioned study progress data and safety data by treatment group. The unblinded closed report will be prepared by the independent statistician (statistical data analyst), and will also be distributed to the DSMB at least 2 weeks prior to the scheduled meeting. The DSMB will additionally be provided with the monitor report(s).

Ad hoc data summaries may be prepared upon request by the DSMB to address a specific safety concern.

6.1 Independent statisticians

As the independent statistician (or statistical data analyst) reporting the (unblinded) data to the DSMB is not a member of the study group, all efforts will be made by the Principal Investigator to ensure both the independent statistician is familiar with the design, setting, objectives of the study, and has access to the database to provide insightful analyses responsive to the DSMB’s needs.

**7. Decision making**

After considering the information in the interim report, the DSMB could give the following recommendations:

1. continue the study according to the study protocol
2. continue the study with modifications to conduct or design
3. discontinue the study due to clear harm
4. discontinue the study due to no effect.

The justifications for a recommendation to terminate the study due to clear harm will be based on data showing a notable increase of (serious) adverse events in the intervention group. No pre-specified formal statistical stopping rule for safety is formulated.

The recommendation to discontinue the study due to no effect is based on recommendations by Freidlin et al., (2010) (1), the DSMB will have a first interim monitoring moment once 25% of information is collected (t1: ‘harm look’). Here, inefficacy monitoring cannot be carried out given the size of the data, but it will be evaluated whether it is likely that the intervention leads to worse outcome than control (77). At 50% (t2) and 75% (t3) of data completion, inefficacy monitoring will be carried out, with the decision boundary being that the 95%CI of the treatment effect does not overlap with the effect that was expected in the trial design under the alternative hypothesis (intervention works better than control). The cut-offs for the expected effects will be set at 0 for t2 to guard against over-aggressive testing. At t3, the cut-off will be set at a 20% more strict cut-off, according to formulae provided by Freidlin et al. (2010)

1. **DSMB report(s) to the Principal Investigator**

Following the DSMB meeting, the DSMB will send a confidential report to the Principal Investigator within 2 weeks after the meeting. The report does not include unblinded data and contains sufficient information to explain the rationale behind any specific recommendation by the DSMB. If no recommendations are made, the report will simply state: ‘The DSMB recommends that the study continues as planned.’ If the Principal Investigator accepts the recommendations, he/she will be responsible for implementing the concerning actions. If the Principal Investigator rejects (parts of the) the DSMB’s recommendations, he/she will provide the DSMB with a written explanation of his decision and supporting rationale within 10 working days. If the DSMB has recommended that the study should be stopped but the Principal Investigator decides to continue the study, the investigator will inform immediately the METC and all concerned regulatory authorities of its decision to continue the study despite the DSMB’s recommendations.

**References**

- 1. Freidlin B, Korn EL, Gray R. A general inefficacy interim monitoring rule for randomized clinical trials. Clinical Trials. 2010;7(3):197–208.
