# Supplementary material for: The Ketamine Trial for Acute Suicidality (KETA): Study Protocol of a Double‐Blind Randomized Placebo‐Controlled Superiority Trial on Intranasal Racemic Ketamine Compared to the Active Placebo Intranasal Midazolam as Treatment for Acute Suicidality
Source: Int J Methods Psychiatr Res. 2025 Nov 19;34(4):e70044. doi: 10.1002/mpr.70044 (PMC12627964; doi:10.1002/mpr.70044)
Supplement: Supplementary file 2 — Supporting Information S2 [file MPR-34-e70044-s001.docx]

**Supplement 2 Laboratory**

| Timepoint (minutes) | Samples (total) | KETA-study | KETA-biobank |
| --- | --- | --- | --- |
| T=-15  (15 min prior to IMP administration) | 2 6 ml EDTA samples | 1 6 ml EDTA sample (BDNF plasma concentration, remaining blood to biobank) | 1 6 ml EDTA sample (4 1 ml aliquots) |
|  | 1 10 ml serum gel sample (1.5ml gel, 8.5 ml blood) | 1/2  10 ml serum gel sample  (fatty acid spectrum, BDNF serum  concentration, remaining blood to biobank) | 1/2 10 ml serum gel sample (4 1 ml aliquots) |
|  | 1 10 ml EDTA sample |  | 1 10 ml EDTA sample (genetics) |
| 180 (+/- 15 minutes) | 2 6 ml EDTA samples | 1 6 ml EDTA sample  (ketamine concentration, BDNF plasma concentration, remaining blood to biobank) | 1 6 ml EDTA sample (4 1 ml aliquots) |
|  | 1 10 ml serum gel sample (1.5 ml gel, 8.5 ml blood) | 1/2 10 ml serum gel sample (BDNF serum concentration, remaining blood to biobank) | 1/2 10 ml serum gel sample (4 1 ml aliquots) |
