# Supplementary material for: The Ketamine Trial for Acute Suicidality (KETA): Study Protocol of a Double‐Blind Randomized Placebo‐Controlled Superiority Trial on Intranasal Racemic Ketamine Compared to the Active Placebo Intranasal Midazolam as Treatment for Acute Suicidality
Source: Int J Methods Psychiatr Res. 2025 Nov 19;34(4):e70044. doi: 10.1002/mpr.70044 (PMC12627964; doi:10.1002/mpr.70044)
Supplement: Supplementary file 3 — Supporting Information S3 [file MPR-34-e70044-s002.docx]

**Ketamine Trial for Acute suicidality (KETA) data management plan. V4 (March 2021)**

**DMP title**

**Project Name** Ketamine Trial for Acute suicidality (KETA) Data Management Plan

**Project Identifier** NL7430.042.20

**Grant Title** 537001004

**Principal Investigator / Researcher** Prof. dr. R.A. Schoevers/ Jurriaan Strous/Gijs Roelandt

**Project Data Contact** j.f.m.strous@umcg.nl, 06-23956398

**Description** Suicide is currently one of the three leading causes of death in the

Netherlands in people aged 15-44 and has a substantial impact on families and

society. Nevertheless, to date no evidence based pharmacological intervention for

acute suicidality exists. Subanaesthetic doses of intravenous ketamine have been

shown to immediately resolve depressive symptoms and suicidal ideation in

depressed patients. However, this effect was never investigated for suicidality per se.

The KETA will be a double blind randomized placebo controlled trial in 112 patients presenting with acute suicidality regardless of the underlying diagnosis, to test the hypothesis that a single dose of 75mg intranasal ketamine is able to diminish acute suicidal ideation. Additionally, we will examine ketamine’s anti-suicidal mechanism of action by measuring plasma and neuroimaging markers. This study may result into a readily available and easily applicable intervention for the treatment of acute suicidality.

**Data Collection**

**What data will you collect or create?**

Original data will be gathered

-clinical and demographic data

-questionnaire scores

-imaging data

-biological data (blood samples)

**How will the data be collected or created?**

The data will be collected from subjects that are enrolled in the Ketamine Trial

For Acute suicidality (KETA).

**Documentation and Metadata**

**What documentation and metadata will accompany the data?**

The data that will be gathered will be defined in a data dictionary.

The quality controls will be described in a data validation plan.

The procedures for gathering the imaging data (performing the MR-scans) and taking blood samples, will be defined.

**Ethics and Legal Compliance**

**How will you manage any ethical issues?**

Consent for data preservation and sharing will be obtained via the Informed Consent Form. The participant's identity will be protected by coding of the data sets.

**How will you manage copyright and Intellectual Property Rights (IPR) issues?**

The University Medical Center Groningen (UMCG) will be the owner of the data.

Data can be requested by third parties if that party intends to use it for answering

research questions. Our research group will assess those requests on quality and

rationale before data will be provided to those parties.

Subjects will be asked beforehand whether the UMCG is allowed to share data with third parties.

**Storage and Backup**

**How will the data be stored and backed up during the research?**

Data will be stored in RedCap, a qualified eCRF system. Redcap uses a

certified data center. When data are to be analysed, data will be stored on a central server in the UMCG. Backups will be made automatically, and are guaranteed by the UMCG Data service.

**How will you manage access and security?**

Both the eCRF system the initial phase (before analysis) and the UMCG for the later phase (during and after analysis), provide an environment for easily accessible and safe long-term preservation of data.

**Selection and Preservation**

**Which data are of long-term value and should be retained, shared, and/or**

**preserved?**

All data, including the raw imaging data have long-term value and will, in principle, be preserved for 15 years. When the most important analyses have been performed and published, we will decide whether the raw imaging data need to be preserved for the full 15 years.

**What is the long-term preservation plan for the dataset?**

After our main analyses have been performed, the data will be uploaded, if the policy

of the UMCG allows us to do so, to a central data repository.

**Data Sharing**

**How will you share the data?**

Data will be shared by means of a central data repository with a unique identifier for our data set. Before sharing we will assess the sharing request on both quality and rationale. We have not made a decision yet on which data repository will be used. We will – if possible - follow the UMCG's policy.

The acquired data will be stored in a data catalogue such as www.zorggegevens.nl. In order to describe our dataset, a metadata scheme by Datacite will be used. Furthermore the DOI-code will be used as a persistent identifier.

**Are any restrictions on data sharing required?**

With regard to accessibility of the data, a restricted access policy will be followed.

Data will only be shared after we have performed our main analyses and after the main results of the study have been published.

Also the rationale for the request needs to be reasonable and the request should be of substantial quality.

**Responsibilities and Resources**

**Who will be responsible for data management?**

J. Strous and G. Roelandt, coordinating investigators (under supervision of Principal Investigator Prof. dr. R.A. Schoevers).

**What resources will you require to deliver your plan?**

The coordinating investigator will need to be trained in the use of the data

management system (RedCap).

Furthermore, we will make use of the UMCG's core facilities for data storage and

Management and the UMCG biobank.
