# Supplementary material for: The Ketamine Trial for Acute Suicidality (KETA): Study Protocol of a Double‐Blind Randomized Placebo‐Controlled Superiority Trial on Intranasal Racemic Ketamine Compared to the Active Placebo Intranasal Midazolam as Treatment for Acute Suicidality
Source: Int J Methods Psychiatr Res. 2025 Nov 19;34(4):e70044. doi: 10.1002/mpr.70044 (PMC12627964; doi:10.1002/mpr.70044)
Supplement: Supplementary file 4 — Supporting Information S4 [file MPR-34-e70044-s006.docx]

**Supplement 4**

**Organizational Structure and responsibilities**

***Steering Committee (SC)***

RS is the lead investigator of the study and head of the SC

JS and GR are the coordinating investigators

JD is the daily supervisor of JS

JK and RM are the daily supervisors of GR.

**Trial Management Committee (TMC)**

RS is the head of the TMC

JS and GR oversee recruitment and data accrual and accrue data

Madelen Moes takes part in data accrual

Martijn Godschalk and Hidde Kleijer occassionaly accrue data.

**Data Management**

Data management is performed by GR and JS, with the aid of the Clinical Research Office of the UMCG. See Supplement for the Data Management Plan.

**Randomization**

The randomized treatment allocation has been generated with ALEA by W. Sloof.

**Funding**

The study is funded by ZonMw grant # 537001004 of the suicide prevention programme. The grant application underwent an independent review process prior to awarding of the funding. This funding source had no role in the design pf the study, and will have no role in the execution, analysis or decisions in regard to submission of the results.

**Availability of data and material**

Researchers interested in the data can contact the first author. Data can be made available upon reasonable request.

**Ethics approval and consent to participate**

Before inception of the study all relevant documents have been submitted to the ethical review board of the University Medical Center Groningen (METC 2020/378), which concluded that the project is in line with the Dutch law (NL74304.042.20). The KETA-study has been registered at the EU Clinical Trial Register (EudraCT 2020-002905-24).

All patients receive both oral and written information about the study. This information comprises the scope and relevance of the study, as well as the patient burden and confidentiality issues. Participants have the possibility to ask research staff for clarification of questions regarding the study. Before participation, written informed consent is obtained at least one hour after patients have been informed in written and oral form.

Substantial amendments will be subtmitted to the Institutional Review Board of the UMCG. All researchers and recruitment sites will be notified.

**Competing Interests**

The authors declare that they have no competing interests.

**Trial Sponsor**

University Medical Center Groningen

Contact name: Prof. dr. Robert Schoevers, [bestuurssecretariaatucp@psy.ucp.nl](mailto:bestuurssecretariaatucp@psy.ucp.nl)

Telephone: +31-(0)50-3616161

Address: Hanzeplein 1, 9713GZ Groningen

**Post Trial Care**

Subjects that may suffer harm are covered by insurance. See supplement for details

**Dissemination of results**

Results of the study will be depicted in several scientific articles. One of them about the results of the trial, one article about blood based biomarkers and one about MRI data. Further articles may arise from the accrued data. In addition, presentations will be given at conferences and results will be disseminated through regular media.

**Revision chronology**

**20 May 2021, Original**

**3 May 2022, Amendment 01. Primary reason for amendment: changes in study protocol after completion of pilot study, detailed in section *feasibility pilot study***
