# Supplementary material for: The Ketamine Trial for Acute Suicidality (KETA): Study Protocol of a Double‐Blind Randomized Placebo‐Controlled Superiority Trial on Intranasal Racemic Ketamine Compared to the Active Placebo Intranasal Midazolam as Treatment for Acute Suicidality
Source: Int J Methods Psychiatr Res. 2025 Nov 19;34(4):e70044. doi: 10.1002/mpr.70044 (PMC12627964; doi:10.1002/mpr.70044)
Supplement: Supplementary file 5 — Supporting Information S5 [file MPR-34-e70044-s005.pdf]

Informatiebrief voor deelnemers aan het onderzoek ‘Intranasale ketamine voor de behandeling van acute suïcidaliteit’ – samenvatting

Geachte heer/mevrouw,

Dit is een samenvatting van de proefpersoneninformatie m.b.t. het onderzoek ‘Intranasale ketamine voor de behandeling van acute suïcidaliteit.’ Op de pagina’s na de samenvatting (vanaf pagina 2) vindt u de volledige informatie.

U bent gevraagd aan dit onderzoek deel te nemen omdat u last heeft van suïcidale gedachten of neigingen. Tot op heden bestaan er geen geneesmiddelen die in staat zijn deze gevoelens snel te doen afnemen. Zoals u zelf waarschijnlijk heeft ervaren, lijden mensen erg onder hun suïcidale gedachten of neigingen, net als hun familie en vrienden. Daarom vinden wij het erg belangrijk dat er een oplossing komt. In een aantal onderzoeken dat de afgelopen jaren verricht is, blijkt ketamine – een pijnstillend en verdovend middel voor operaties dat sinds 1970 op de markt is – een krachtig en zeer snel antidepressief effect te hebben. Omdat het zo snel tegen depressie werkt, zou het misschien ook snel suïcidale gevoelens kunnen verminderen.

Als u aan de studie deel wilt nemen, krijgt u neusspray toegediend met ofwel ketamine ofwel een placebo (midazolam, een stof die net als ketamine slaapverwekkend kan werken). Zowel u als de arts weten niet wat u krijgt, dit is van belang voor de kwaliteit van het onderzoek. Voordat u de ketamine of het placebo heeft gekregen, en 1 en 3 uur daarna nemen we een aantal vragenlijsten bij u af die de mate van suïcidaliteit, depressie en eventuele bijwerkingen meten. U verblijft tot de eerstvolgende ochtend na inname van het medicijn op onze psychiatrische medische unit. We zullen u vervolgens 1 en 3 dagen en 1, 2 en 4 weken nadat u de neusspray toegediend gekregen heeft bellen om deze vragenlijsten nog een keer af te nemen.

Om een beter inzicht te krijgen in de vraag hoe ketamine werkt, zullen we bloed bij u afnemen voordat u de neusspray krijgt en 3 uur daarna.

Ook zouden we in het geval dat in Groningen aan de studie deelneemt, als u daarmee instemt, 1 dag na toediening van de neusspray een MRI-scan van uw hersenen willen maken om beter te kunnen begrijpen hoe ketamine werkt.

U kunt alleen aan het onderzoek deelnemen als u de toestemmingsverklaring heeft getekend.

Hierna volgt de meer uitgebreide informatie.

Informatiebrief voor deelnemers aan het onderzoek
'Intranasale ketamine voor de behandeling van acute suïcidaliteit'

Geachte heer mevrouw,

Wij vragen u vriendelijk om deel te nemen aan een medisch-wetenschappelijk onderzoek. Meedoen is vrijwillig. Om mee te doen is wel uw schriftelijke toestemming nodig. U ontvangt deze informatiebrief omdat u last heeft van ernstige zelfmoordneigingen, oftewel acute suïcidaliteit. Voor acute suïcidaliteit is tot op heden geen werkzame medicatie gevonden. Wij willen onderzoeken of ketamine een antisuïcidaal effect heeft. Wij vragen u daarom om deel te nemen aan dit onderzoek. Of u deel wilt nemen aan het onderzoek is beslist u zelf. Voordat u beslist of u mee wilt doen is het van belang dat u meer over het onderzoek te weten komt. In deze brief zullen wij de inhoud van het onderzoek zo begrijpelijk mogelijk beschrijven.

Wij vragen u deze brief goed te lezen, en zo nodig te bespreken met uw partner of familieleden. Indien u besluit niet aan het onderzoek deel te nemen, dan heeft dit geen gevolgen voor uw verdere behandeling. Indien u na het lezen van deze informatiebrief nog vragen heeft, dan kunt u terecht bij de aanwezige onderzoeker of dienstdoende arts.

Dit onderzoek wordt in verschillende ziekenhuizen uitgevoerd, in het Universitair Medisch Centrum Groningen (UMCG) en bij de GGz-instelling Lentis in Groningen. Voor dit onderzoek zijn 100 proefpersonen nodig. De medisch ethische toetsingscommissie heeft dit onderzoek goedgekeurd. Algemene informatie over de toetsing van het onderzoek vindt u in de brochure 'Medisch-wetenschappelijk onderzoek.'

Doel van het onderzoek

Het doel van dit onderzoek is uitzoeken hoe veilig en werkzaam het medicijn ketamine is voor de behandeling van acute suïcidaliteit. Ketamine is al sinds 1970 op de markt, maar wordt nu alleen gebruikt als verdovingsmiddel bij operaties en bij pijnbestrijding. Uit een aantal onderzoeken is gebleken dat ketamine helpt tegen depressiviteit en mogelijk ook tegen suïcidaliteit.

De werking van ketamine vergelijken we met de werking van een ander medicijn als placebo: midazolam. Midazolam is een slaapverwekkend medicijn dat we in dit onderzoek gebruiken als placebo omdat het geen effect heeft op suïcidaliteit maar wel een vergelijkbaar rustgevend gevoel geeft als ketamine. Zo kunt u niet weten of u ketamine of placebo gekregen heeft. Dit is van belang voor het slagen van het onderzoek.

Wat meedoen inhoudt

Als u meedoet, duurt dat, inclusief het afnemen van alle vragenlijsten, een week. U krijgt de ketamine of midazolam éénmalig toegediend via een neusspray

Keuring
Eerst bepalen we of u kunt meedoen. De onderzoeker bepaalt of u boven een bepaalde waarde scoort op een suïcidaliteitsvragenlijst. Ook doet hij een algemeen lichamelijk onderzoek en meet hij gewicht, bloeddruk, temperatuur en ademhalingsfrequentie en zal hij u een aantal vragen stellen over uw medische en psychiatrische voorgeschiedenis. U kunt niet deelnemen aan het onderzoek als u:
-een ernstige lichamelijke medische aandoening heeft.
-onder invloed bent van GHB of een bloed-alcoholconcentratie van boven de 0,05% heeft.
-schizofrenie heeft of een andere primaire psychotische stoornis.
-bekend bent met een misbruik van ketamine of een overgevoeligheid voor ketamine.
-zwanger bent of - indien u vrouw bent en heteroseksueel actief bent, geen betrouwbare anticonceptie gebruikt. Om zwangerschap uit te sluiten wordt een zwangerschapstest gedaan.
-neuspoliepen heeft of een (ernstige) neusverstopping heeft.
-eerder aan deze studie deelgenomen heeft.
-een MAO-remmer (een specifiek type antidepressivum) gebruikt
-bepaalde medicijnen die een wisselwerking met ketamine of midazolam kunnen hebben, gebruikt

Behandeling
We behandelen u éénmalig met een neusspray. De helft van de proefpersonen krijgt ketamine-neusspray (75mg), de andere helft krijgt midazolam-neusspray (4,0mg). Loting bepaalt welke behandeling u krijgt. U en de onderzoeker weten niet in welke groep u zit. Als dit voor uw gezondheid belangrijk is, kan dit wel worden opgezocht.

Metingen en verrichtingen
-Een kwartier voordat u de neusspray krijgt zullen vier buisjes bloed, en drie uur nadat u de neusspray gekregen heeft, zullen drie buisjes bloed bij u afgenomen worden. In totaal nemen we 54 ml bloed bij u af.
-Net voordat u de neusspray krijgt en 1 en 3 uur erna zullen 4 vragenlijsten bij u afgenomen worden, per keer zal dit zo'n 10 minuten duren:
-de BSSI, een suïcidaliteitsvragenlijst.
-de MADRS: een depressievragenlijst,
-de SAFTEE, een vragenlijst over bijwerkingen.
-Nadat u de neusspray toegediend heeft gekregen zult u tot de volgende ochtend in het ziekenhuis blijven ter observatie. Vervolgens kunt u onder begeleiding van een bekende naar huis tenzij uw behandelend arts het noodzakelijk vindt u op te nemen.
-1 en 3 dagen en 1, 2 en 4 weken nadat u de neusspray toegediend heeft gekregen, zult u door de onderzoeker gebeld worden, en zullen bovenstaande vragenlijsten opnieuw, ditmaal telefonisch afgenomen worden. Ook zullen we op 1 dag na de neusspray de MINI-vragenlijst en de early childhood

traumavragenlijst afnemen. Dit zal op die dag bij elkaar ongeveer een half uur duren.
-Indien u dat wilt, en u deelnemer in Groningen bent, zal een dag na toediening van de ketamine of midazolam een MRI-scan van uw hersenen gemaakt worden. Dit is aansluitend aan de opname, u hoeft hiervoor dus niet opnieuw naar het ziekenhuis te komen. We willen deze scans maken om een beter idee te krijgen van het precieze effect dat ketamine op de hersenen heeft. Bij deze scans komt geen straling vrij, wel kan de scanner een hard geluid maken en is de ruimte waarin u moet liggen klein. Het is belangrijk dat u geen metalen objecten in uw lichaam heeft als u aan dit onderdeel van het onderzoek mee wilt doen. Wij beoordelen de MRI-scan in het kader van de onderzoeksvraag van dit wetenschappelijke onderzoek. Hoewel we niet zoeken naar andere afwijkingen, komt het wel eens voor dat we iets zien dat ongebruikelijk is. In dat geval laten we de MRI-scan door een deskundige beoordelen, en nemen, als het een afwijking betreft die met u besproken moet worden contact met u en uw huisarts op. Als u niet geïnformeerd wilt worden over afwijkende bevindingen of u het er niet mee eens bent dat u huisarts hiervan op de hoogte gesteld wordt, kunt u niet deelnemen aan dit (deel van) het onderzoek.

Anders dan gebruikelijke zorg
Normaalgesproken bespreekt de dienstdoende psychiater met u waarom u suïcidaal bent, en wat daaraan gedaan zou kunnen worden. Deze gesprekken zullen nog steeds plaatsvinden, echter krijgt u er nu eenmalig ketamine of midazolam via de neusspray bij. Er bestaat nog geen medicamenteuze behandeling voor suïcidaliteit, dus de neusspray is geen vervanging van een gebruikelijke medicamenteuze behandeling.
U krijgt de neusspray slechts eenmalig toegediend. Vervolgens zal de zorg doorgaan op de gebruikelijke wijze, zoals dat ook gegaan zou zijn als u niet aan dit onderzoek deelgenomen zou hebben. Wel wordt u in het kader van dit onderzoek tot en met de volgende ochtend na de toediening van de neusspray opgenomen. Uw behandelend arts beslist of u daarna nog in het kader van de reguliere zorg zou moeten blijven. Gedurende uw gehele verblijf valt u onder de medische verantwoordelijkheid van uw behandelend arts.

Welke afspraken zijn van belang?
Om het onderzoek goed te doen en voor uw eigen veiligheid, zijn, als u ervoor kiest om deel te nemen, is het van belang dat u:

-de onderzoekers toelaat de medicatie toe te dienen.
-op het moment van dit onderzoek niet aan een ander medisch-wetenschappelijk onderzoek deelneemt.
-u beschikbaar bent voor telefonisch contact op de afgesproken tijdstippen.
-u de deelnemerskaart van het onderzoek bij u draagt. Hierop staat dat u meedoet aan dit onderzoek. Er staat ook op wie in geval van nood moet worden gewaarschuwd. We verzoeken u deze kaart te laten zien als u tijdens het onderzoek bij een andere arts komt.

Het is belangrijk dat u, tijdens de duur van het onderzoek, contact opneemt met de onderzoeker
-voordat u (tijdens de duur van het onderzoek) andere geneesmiddelen gaat gebruiken, ook als dat homeopathische geneesmiddelen, natuurgeneesmiddelen, vitaminen en/of geneesmiddelen van de drogist zijn.
-als u in een ziekenhuis wordt opgenomen of behandeld.
-als u plotseling gezondheidsklachten krijgt
-als u niet meer wilt meedoen aan het onderzoek
-als uw contactgegevens wijzigen.

Zwangerschap van u of uw partner
Vrouwen die zwanger zijn of borstvoeding geven, kunnen niet meedoen aan dit onderzoek.

Mogelijke bijwerkingen/complicaties en andere nadelige effecten/ongemakken
De volgende voorbijgaande bijwerkingen kunnen voorkomen:
-U kunt tijdelijk vreemde dingen gaan zien of horen (visuele- of geluidshallucinaties).
-U kunt tijdelijk een gevoel van vervreemding; 'dissociatie' krijgen,
-Verder kunnen de volgende bijwerkingen optreden: verwardheid, vertraagde of versnelde hartslag, misselijkheid, verminderde eetlust, een droge mond en slaperigheid.
-Verandering in de bloeddruk
-Het maken van de MRI-scans: u ligt in een kleine ruimte en hoort harde geluiden.
-Er bestaat een risico op het optreden van ketaminemisbruik; het teveel en op een ongezonde wijze gebruiken van de stof.

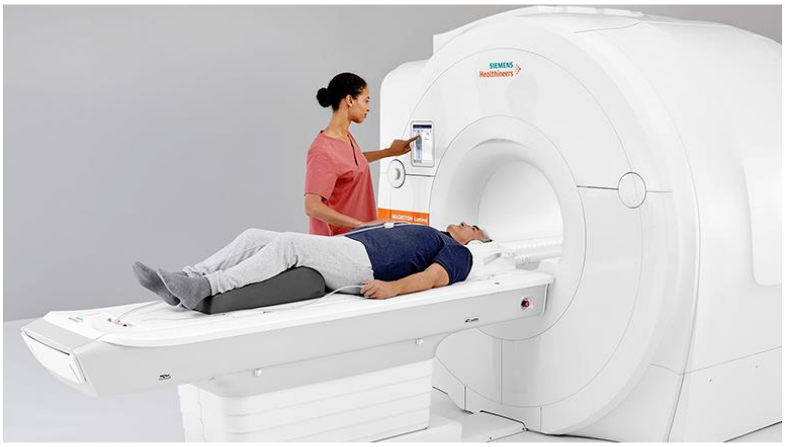

een afbeelding van de MRI-scanner www.medgadget.com

Metingen
Bloedafnames kunnen pijn doen of een bloeduitstorting geven. Alles bij elkaar nemen we 54 ml bloed bij u af. Deze hoeveelheid geeft bij volwassenen geen problemen. Ter vergelijking: bij de bloedbank wordt 500ml per keer afgenomen.

De concentratie van de ketamine, van vetzuren en een groeifactor voor zenuwcellen zal bepaald worden.

Mogelijke voor- en nadelen
Mogelijke voordelen van deelname aan dit onderzoek:
-De onderzoeksmedicatie kan tot een vermindering van uw suïcidale en depressieve gevoelens leiden, maar zeker is dat niet. Op elk moment tijdens het onderzoek kunnen uw klachten aanwezig blijven, terugkomen of verslechteren
-Deelname aan dit onderzoek betekent dat u een bijdrage levert aan de ontwikkeling van medische kennis waar medepatiënten (en eventueel uzelf) mogelijk voordeel van hebben.
-Wij zullen u op de hoogte stellen van eventuele ernstige aandoeningen die we het bij MRI-onderzoek vinden. Als u hier niet van op de hoogte gesteld wilt worden, kunt u niet deelnemen aan dit onderdeel van het onderzoek.

Mogelijke nadelen van deelname aan dit onderzoek:
-extra tijd die het u kost
-extra testen (bloedafname, vragenlijsten).
-het maken van de MRI-scans: u ligt in een kleine ruimte en hoort harde geluiden.
-afspraken waaraan u zich moet houden
-mogelijke bijwerkingen (zoals eerder omschreven)
-mogelijke ongemakken van de bloedafname, zoals pijn of een blauwe plek.
-het risico op ketaminemisbruik

Als u niet wilt meedoen of wilt stoppen met het onderzoek
U beslist zelf of u meedoet aan het onderzoek. Deelname is vrijwillig. Mocht u besluiten niet deel te willen nemen, wordt u volgens de richtlijnen behandeld voor uw suïcidaliteit. De onderzoeker kan u meer vertellen over de mogelijkheden die er zijn en de voor- en nadelen daarvan.

Doet u wel mee aan het onderzoek, dan kunt u zich altijd bedenken. U mag tijdens het onderzoek stoppen. U wordt dan weer op de gebruikelijke manier behandeld voor uw suïcidaliteit. U hoeft niet te zeggen waarom u stopt. Wel moet u dit direct melden aan de onderzoeker.

De gegevens die tot dat moment zijn verzameld, worden gebruikt voor het onderzoek. Als u wilt, kan verzameld materiaal worden vernietigd.

Als er nieuwe informatie over het onderzoek is die belangrijk voor u is, laat de onderzoeker dit aan u weten. U wordt gevraagd of u blijft meedoen.

Einde van het onderzoek
Uw deelname aan het onderzoek stopt als
-uw ziekenhuisbezoek en de telefonische afspraken voorbij zijn.
-u zelf kiest om te stoppen.

-het einde van het onderzoek is bereikt
-de onderzoeker het beter voor u vindt om te stoppen
-de ethische toetsingscommissie, de overheid of het Universitair Medisch Centrum Groningen besluit om het onderzoek te stoppen

Het hele onderzoek is afgelopen als alle deelnemers klaar zijn. De medicatie die u gebruikt heeft bij het onderzoek, is niet beschikbaar na afloop van het onderzoek. De onderzoeker zal met u praten over de mogelijkheden voor verdere medische zorg.

Na verwerken van alle gegevens informeert de onderzoeker u over de belangrijkste uitkomsten van het onderzoek. Dit gebeurt ongeveer vier jaar na uw deelname.

De onderzoeker kan u dan ook vertellen in welke groep u zat (ketamine of midazolam). Als u dit niet wilt, dan kunt u dit tegen de onderzoeker zeggen. Hij mag het u dan niet vertellen.

Gebruik en bewaren van uw gegevens en lichaamsmateriaal
Voor dit onderzoek worden uw persoonsgegevens en lichaamsmateriaal verzameld, gebruikt en bewaard. Het gaat om gegevens zoals uw naam, adres, geboortedatum en om gegevens over uw gezondheid. Voor dit onderzoek is het nodig dat uw bloed wordt verzameld en gebruikt.
De gegevens worden 15 jaar bewaard voor ander onderzoek, u geeft daar op het bijgaande toestemmingsformulier wel of geen toestemming voor.
Uw bloed zal in een biobank opgeslagen worden, hiervoor kunt u op een apart formulier wel of geen toestemming geven.

Toegang tot uw gegevens voor controle
Sommige personen kunnen op de onderzoekslocatie toegang krijgen tot al uw gegevens. Ook tot de gegevens zonder code. Dit is nodig om te kunnen controleren of het onderzoek goed en betrouwbaar is uitgevoerd. Personen die ter controle inzage krijgen in uw gegevens zijn:
-de commissie die de veiligheid van het onderzoek in de gaten houdt
-een monitor die voor de opdrachtgever van het onderzoek werkt
-nationale toezichthoudende autoriteiten, bijvoorbeeld de Inspectie Gezondheidszorg
-de apotheek, die de studiemedicatie labelt en uitzet

Zij houden uw gegevens geheim. Wij vragen u voor deze inzage toestemming te geven.

Bewaartermijn gegevens en lichaamsmateriaal
Uw gegevens moeten 15 jaar worden bewaard op de onderzoekslocatie. Uw bloed zal worden gebruikt voor het bepalen van de hoeveelheid en ketamine en de hoeveelheid *Brain Derived Neurotrophic Factor (BDNF)*, een groeifactor voor zenuwcellen. Wij denken dat zenuwcellen bij suïcidaliteit beschadigd zijn, en dat

BDNF deze schade mogelijk tijdelijk kan verminderen, waardoor de suïcidale gevoelens af zouden kunnen nemen.
Als u toestemming geeft, bewaren we het bloed om daarop in de loop van dit onderzoek nog nieuwe bepalingen te kunnen doen die te maken hebben met het onderzoek. Zo zouden we in uw bloed ook de concentraties van vetzuren willen meten; deze stoffen zijn van belang voor het onderhoud van zenuwcellen.

Bewaren en gebruik van gegevens en lichaamsmateriaal voor ander onderzoek
Uw gegevens en lichaamsmateriaal kunnen na afloop van dit onderzoek ook nog van belang zijn voor ander wetenschappelijk onderzoek op het gebied van suïcidaliteit. Daarvoor zullen uw gegevens 15 jaar worden bewaard en uw lichaamsmateriaal 15 jaar worden bewaard in een biobank. U kunt op het toestemmingsformulier aangeven of u hier wel of niet mee instemt. Indien u hier niet mee instemt, kunt u gewoon deelnemen aan het huidige onderzoek.

Meer informatie over uw rechten bij verwerking van gegevens
Voor algemene informatie over uw rechten bij verwerking van uw persoonsgegevens kunt u de website van de Autoriteit Persoonsgegevens, het UMCG of Lentis raadplegen.

Bij vragen over uw rechten kunt u contact opnemen met de verantwoordelijke voor de verwerking van uw persoonsgegevens. Voor dit onderzoek is dat:

Prof. dr. R.A. Schoevers. Zie bijlage A voor contactgegevens en website.

Bij vragen of klachten over de verwerking van uw persoonsgegevens raden we u aan eerst contact op te nemen met de onderzoekslocatie. U kunt ook contact opnemen met de Functionaris voor de Gegevensbescherming van de instelling, Piet Dinjens of Boudien Sieperda (beiden UMCG), de functionaris bij Lentis (zie voor de contactgegevens bijlage A) of de Autoriteit Persoonsgegevens.

Registratie van het onderzoek
Dit onderzoek staat in een overzicht van medisch-wetenschappelijke onderzoeken, namelijk *het Nederlands Trial Register* www.trialregister.nl. Deze website bevat geen informatie die herleidbaar is tot u als persoon. Wel kan de website een samenvatting van de resultaten tonen. U vindt dit onderzoek onder *NL8873.*

Verzekering proefpersonen
Wij hebben voor iedereen die aan dit onderzoek deelneemt een verzekering afgesloten. Deze verzekering dekt alle schade die als gevolg van het onderzoek ontstaat. Dit geldt voor schade tijdens en binnen vier jaar na het onderzoek. Zie voor meer informatie bijlage C.

Informeren huisarts en/of behandelend specialist en/of apotheker
Wij sturen uw huisarts, uw ambulant behandelaar en/of behandelend specialist en/of apotheker altijd een brief/e-mail om te laten weten dat u meedoet aan het onderzoek. Dit is voor uw eigen veiligheid. Als u dit niet goed vindt, kunt u niet meedoen aan dit onderzoek. U kunt niet deelnemen aan het onderzoek als u geen huisarts heeft.

Geen vergoeding voor meedoen
De studiemedicatie voor het onderzoek kost niets. U wordt niet betaald voor het meedoen aan dit onderzoek.

Heeft u vragen?
Bij vragen kunt u contact opnemen met het onderzoeksteam. Voor onafhankelijk advies over meedoen aan dit onderzoek kunt u terecht bij de onafhankelijk arts, Sjoerd van Belkum (UMCG). Hij weet veel over het onderzoek, maar heeft niets te maken met dit onderzoek.

Indien u klachten heeft over het onderzoek, kunt u dit bespreken met de onderzoeker of uw behandelend arts. Wilt u dit liever niet, dan kunt u zich wenden tot de klachtencommissie van het ziekenhuis. Alle gegevens vindt u in **bijlage A:** Contactgegevens.

Ondertekening toestemmingsformulier
Gezien het acute karakter van de studie heeft u slechts een uur om te bedenken of u deel wilt nemen aan dit onderzoek. Indien u toestemming geeft, zullen wij u vragen deze op de bijbehorende toestemmingsverklaring schriftelijk te bevestigen. Door uw schriftelijke toestemming geeft u aan dat u de informatie heeft begrepen en instemt met deelname aan het onderzoek. Zowel uzelf als de onderzoeker ontvangen een getekende versie van deze toestemmingsverklaring.

Dank voor uw aandacht.

Bijlagen bij deze informatie

A. Contactgegevens
B. Informatie over de verzekering
C. Schema onderzoekshandelingen
D. Toestemmingsformulier
E. Brochure 'Medisch-wetenschappelijk onderzoek. Algemene informatie voor de proefpersoon.'

Bijlage A.1 : contactgegevens voor Universitair Medisch Centrum Groningen

Hoofdonderzoeker:
Prof. dr. Robert Schoevers
050-3612065
bestuurssecretariaatucp@umcg.nl (mailadres van secretaresse Heleen Mellies)

Uitvoerend onderzoekers:
Jurriaan Strous
050-3616161
j.f.m.strous@umcg.nl
Gijs Roelandt
050-3616161
g.h.j.roelandt@umcg.nl

Onafhankelijk arts:
Dr. Sjoerd van Belkum
s.m.van.belkum@umcg.nl

Klachten:
Klachtenfunctionaris UMCG, klachtenfunctionaris@umcg.nl

Functionaris voor de Gegevensbescherming van de instelling:
Piet Dinjens en Boudien Sieperda
privacy@umcg.nl

Website Autoriteit Persoonsgegevens:
www.autoriteitpersoonsgegevens.nl

Website persoonsgegevens UMCG:
Website t.a.v. privacy UMCG
https://www.umcg.nl/NL/UMCG/Paginas/Privacy-statement-UMCG.aspx

Bijlage A.2 : contactgegevens voor Lentis

Uitvoerend onderzoeker:
Jurriaan Strous
050-3616161
j.strous@lentis.nl

Onafhankelijk arts:
Dr. Sjoerd van Belkum
050-3616161
s.m.van.belkum@umcg.nl

Klachten:
Klachtenfunctionaris Lentis
klachtenfunctionaris@lentis.nl

Functionaris voor de gegevensbescherming van de instelling
fg@lentis.nl

Website Autoriteit Persoonsgegevens:
www.autoriteitpersoonsgegevens.nl

Bijlage B: informatie over de verzekering

Voor iedereen die meedoet aan dit onderzoek, heeft de verrichter een verzekering afgesloten. De verzekering dekt schade door deelname aan het onderzoek. Dit geldt voor schade tijdens het onderzoek of binnen vier jaar na het einde ervan. Schade moet u binnen die vier jaar aan de verzekeraar hebben gemeld.

De verzekering dekt niet alle schade. Onderaan deze tekst staat in het kort welke schade niet wordt gedekt.
Deze bepalingen staan in het Besluit verplichte verzekering bij medisch-wetenschappelijk onderzoek met mensen. Dit besluit staat op www.ccmo.nl, de website van de Centrale Commissie Mensgebonden Onderzoek (zie 'Bibliotheek' en dan 'Wet- en regelgeving').

Bij schade kunt u direct contact leggen met de verzekeraar [of schaderegelaar].

De verzekeraar van het onderzoek is:<br>Naam: Centramed<br>Adres: Maria Montessorilaan 9 Zoetermeer<br>Telefoonnummer: 070-3017070<br>E-mail: info@centramed.nl

De verzekering biedt een dekking van 650.000per proefpersoon en 5.000.000 voor het hele onderzoek en 7.500.000 per jaar voor alle onderzoeken van dezelfde opdrachtgever.

De verzekering dekt de volgende schade **niet**:

- schade door een risico waarover u in de schriftelijke informatie bent ingelicht. Dit geldt niet als het risico zich ernstiger voordoet dan was voorzien of als het risico heel onwaarschijnlijk was;
- schade aan uw gezondheid die ook zou zijn ontstaan als u niet aan het onderzoek had meegedaan;
- schade door het niet (volledig) opvolgen van aanwijzingen of instructies;
- schade aan uw nakomelingen, als gevolg van een negatief effect van het onderzoek op u of uw nakomelingen;
- schade door een bestaande behandelmethode bij onderzoek naar bestaande behandelmethoden.

Bijlage C

Schema onderzoekshandelingen

Tijdstip	Actie	Manier van contact
Pre-enrolment (screening)	BSSI - vragenlijst	Face-to-face
-15 minuten	2x 6ml, 1x 10ml en 1x 10ml buis bloed	Face-to-face
0 minuten	ketamineneusspray	Face-to-face
60 minuten	4 vragenlijsten: BSSI, MADRS, SAFTEE, CADSS	Face-to-face
180 minuten	2x 6ml en 1x 10 ml bloed, 3 vragenlijsten	Face-to-face
1 dag	4 vragenlijsten: BSSI, MADRS, MINI, early childhood trauma<br>MRI scan (deelnemers in Groningen)<br>Vraag naar het aantal suïcidepogingen	Face-to-face/Telefonisch
3 dagen	-2 vragenlijsten<br>-Vraag naar het aantal suïcidepogingen	Telefonisch
1 week	-2 vragenlijsten<br>-Vraag naar het aantal suïcidepogingen	Telefonisch
2 weken	-2 vragenlijsten<br>-Vraag naar het aantal suïcidepogingen	Telefonisch
4 weken	-2 vragenlijsten<br>-Vraag naar het aantal suïcidepogingen	Telefonisch

Bijlage D

Toestemmingsformulier voor deelnemers aan het onderzoek 'Effectiviteit van ketamine bij de behandeling van acute suïcidaliteit (Ketamine Trial for Acute suicidality (KETA))'
ToetsingOnline # 74304
versie 28-4-2022

Ik heb de informatiebrief voor de proefpersoon gelezen. Ik kon aanvullende vragen stellen. Mijn vragen zijn genoeg beantwoord. Ik had genoeg tijd om te beslissen of ik meedoe.

Ik weet dat meedoen helemaal vrijwillig is. Ik weet dat ik op ieder moment kan beslissen om toch niet mee te doen. Daarvoor hoef ik geen reden te geven.

Ik geef toestemming voor het informeren van mijn huisarts dat ik meedoe aan dit onderzoek.

Ik geef toestemming om mijn huisarts/ambulante behandelaar/specialist die mij behandelt te vertellen dat ik meedoe aan dit onderzoek.

Ik geef toestemming voor het verzamelen en gebruiken van mijn gegevens en bloedmonsters voor de beantwoording van de onderzoeksvraag in dit onderzoek.

Ik weet dat voor de controle van het onderzoek sommige mensen toegang tot al mijn gegevens kunnen krijgen. Die mensen staan vermeld in de informatiebrief. Ik geef toestemming voor die inzage door deze personen.

Ik geef toestemming om mijn onderzoeksgegevens 15 jaar na afloop van dit onderzoek te bewaren.

Indien bij MRI-onderzoek een aandoening gevonden wordt die ofwel te voorkomen dan wel te genezen of behandelen is, word ik hiervan op de hoogte gesteld.

Ik geef toestemming voor het informeren van mijn huisarts en/of behandelend specialist van onverwachte bevindingen die van belang (kunnen) zijn voor mijn gezondheid.

Ik weet dat ik niet zwanger mag zijn ten tijde van toediening van het onderzoeksmiddel en niet zwanger mag worden tot na twee weken na toediening van het onderzoeksmiddel of placebo.

Ik geef □ **wel**
□ **geen**

toestemming om mijn persoonsgegevens langer te bewaren en te gebruiken voor toekomstig onderzoek op het gebied van suïcidaliteit

Ik geef □ **wel**
□ **geen**

toestemming om deel te nemen aan het MRI-onderzoek.

Ik geef □ **wel**
□ **geen**

toestemming om mij na dit onderzoek opnieuw te benaderen voor een vervolgonderzoek.

Ik wil □ **wel**
□ **niet**

geïnformeerd worden over welke behandeling ik heb gehad/in welke groep ik zat.

Ik wil meedoen aan dit onderzoek.

Naam proefpersoon:

Handtekening: Datum : __ / __ / __

Ik verklaar hierbij dat ik deze proefpersoon volledig heb geïnformeerd over het genoemde onderzoek.

Als er tijdens het onderzoek informatie bekend wordt die de toestemming van de proefpersoon zou kunnen beïnvloeden, dan breng ik hem/haar daarvan tijdig op de hoogte. Naam onderzoeker (of diens vertegenwoordiger):

Handtekening: Datum: __ / __ / __

Aanvullende informatie is gegeven door (indien van toepassing):

Naam:

Functie:

Handtekening: Datum: __ / __ / __

----

* Doorhalen wat niet van toepassing is.
