# Supplementary material for: The Ketamine Trial for Acute Suicidality (KETA): Study Protocol of a Double‐Blind Randomized Placebo‐Controlled Superiority Trial on Intranasal Racemic Ketamine Compared to the Active Placebo Intranasal Midazolam as Treatment for Acute Suicidality
Source: Int J Methods Psychiatr Res. 2025 Nov 19;34(4):e70044. doi: 10.1002/mpr.70044 (PMC12627964; doi:10.1002/mpr.70044)
Supplement: Supplementary file 6 — Supporting Information S6 [file MPR-34-e70044-s007.docx]

|  | **KETA-study WHO-data** | |
| --- | --- | --- |
| **1.** | **Primary Registry and Trial Identifying Number** | EudraCT 2020-002905-24 |
| **2.** | **Date of Registration in Primary Registry** | 6 October 2021 |
| **3.** | **Secondary Identifying Numbers** | METC 2020/378 (Institutional Review Board Universtiy Medical Center Groningen,  NL74304.042.20 (ABR-form, Toetsingonline, a Dutch trial registry). |
| **4.** | **Source(s) of Monetary or Material Support** | Zonmw, grant number # 537001004 (suicide prevention |
| **5.** | **Primary Sponsor** | University Medical Center Groningen (UMCG) |
| **6.** | **Secondary Sponsor(s)** | None |
| **7.** | **Contact for Public Queries** | Jurriaan Strous, j.f.m.strous@umcg.nl |
| **8.** | **Contact for Scientific Queries** | Jurriaan Strous, j.f.m.strous@umcg.nl |
| **9.** | **Public Title** | The Ketamine Trial for Acute suicidality (KETA) |
| **10.** | **Scientific Title** | The Ketamine Trial for Acute suicidality (KETA): Study protocol of a double blind randomized placebo-controlled superiority trial on intranasal racemic ketamine compared to the active placebo intranasal midazolam as treatment for acute suicidality. |
| **11.** | **Countries of Recruitment** | The Netherlands |
| **12.** | **Health Condition(s) or Problem(s) Studied** | Acute suicidality |
| **13.** | **Intervention(s)** | Active comparator: A single 75mg intranasal ketamine administration  Placebo comparator: a single 4mg intranasal midazolam administration |
| **14.** | **Key Inclusion and Exclusion Criteria** | *Inclusion criteria:* an increase in suicidality in the 96 hours before study drug administration, age 18-70.  *Exclusion criteria*: current psychotic episode, schizophrenia or other primary psychotic disorders. |
| **15.** | **Study Type** | Double blind (active) placebo controlled trial. |
| **16.** | **Date of First Enrollment** | 5 October 2022 |
| **17.** | **Sample Size** | 100 |
| **18.** | **Recruitment Status** | Recruiting |
| **19.** | **Primary Outcome(s)** | Change in suicidality |
| **20.** | **Key Secondary Outcomes** | the reduction in SI as measured by the Beck Scale for Suicide Ideation (BSSI) at 180 minutes after administration in the ketamine condition relative to midazolam condition. |
| **21.** | **Ethics Review** | Approved, 7 September 2022 |
| **22.** | **Completion date** | - |
| **23.** | **Summary Results** | - |
| **24.** | **IPD sharing statement** | IPD can be shared upon reasonable request. |
