# Supplementary material for: The Ketamine Trial for Acute Suicidality (KETA): Study Protocol of a Double‐Blind Randomized Placebo‐Controlled Superiority Trial on Intranasal Racemic Ketamine Compared to the Active Placebo Intranasal Midazolam as Treatment for Acute Suicidality
Source: Int J Methods Psychiatr Res. 2025 Nov 19;34(4):e70044. doi: 10.1002/mpr.70044 (PMC12627964; doi:10.1002/mpr.70044)
Supplement: Supplementary file 8 — Supporting Information S8 [file MPR-34-e70044-s008.pdf]

umcg

MONITORING PLAN, 10-11-2021/1.0

Efficacy and feasibility of intranasal ketamine on acute suicidality, a multicenter double blind randomized placebo-controlled trial (Ketamine Trial for Acute suicidality, KETA)

**Completed by**				
Full name	Role in study	Department	Date	Signature
**Hedde Strooisma**	Monitor	SDCRO	10-11-2021	H Strooisma
**Paolo Toffanin**	Monitor (in training)	SDCRO	10-11-2021	Paolo Toffanin

**Authorized by**				
Full name	Role in study	Department	Date	Signature
	Investigator or delegate			

Section 1: General	
Abbreviated study title	KETA
Protocol number/date	Pilot 1.1 / 1 December 2020<br>Main study: 1.3 / 1 December 2020
METc study number	Pilot & Main study: 2020/378
UMCG research register number	Pilot & Main study: 202000511
Principal Investigator	Prof. dr. R.A. Schoevers
Co/sub investigator	Prof. dr. M.W. Hollmann
Contact person	Drs. J.F.M. Strous

Section 2: Specification of documents and procedures	
Protocol	**Pilot***<br>Version: 1.1<br>Date: 1 December 2020<br>Date approval (or expected): 8th June 2021<br>**Main Study***<br>Version: 1.3<br>Date: 1 December 2020<br>Date approval (or expected): 8th June 2021
***NOTE:** The METC approved the main study with the prerequisite that a pilot study will be performed beforehand to determine the feasibility of the study as it was currently set-up by the researchers.	
Risk classification	Moderate risk
Case Report Form (CRF)	eCRF: specify system: RedCap<br>Date and/or version: 10.0.23 / 2021
Approved patient information sheet(s) and informed consent form(s)	**Pilot**<br>Version: 1.0<br>Date: 2 December 2020<br><br>**Main Study**<br>Version: 1.2<br>Date: 2 December 2020

Section 3: Milestones	
Planned date first patient in	***Pilot: 30-09-2021***<br>***Main Study: TBD***
Planned date last patient in	**Pilot:** 30-12-2021<br>**Main Study**: 01-07-2024
Planned date last patient out	**Pilot:**7-1-2022<br>**Main Study:** 08-07-2024
Planned date database lock (if applicable)	Q3-2024

Section 4: Patients and sites to be monitored	
Number of sites and location	2 sites<br>Locations: UMC Groningen and other feasible site*<br>* GGZCentraal Almere is not participating anymore, the researchers are trying to find a new appropriate site
Planned number of patients per site	**Pilot**: 12<br>**Main Study**: 100

Total number of patients in study	4
Current number of patients included	4

Section 5: Specification of non CRF data important for analysis	
Data that is not part of the CRF, but will be collected for the study and need monitoring	All data will be included in the eCRF.

Section 6: Specifications of monitoring visit	
**Planning monitoring**	Frequency of monitor visits: 1 or 2 per year depending on inclusion progress/rate<br>Total visits: 12 *visits: 6 on-site (3 per-site) 6 remote (3 per-site)*<br>Planning first monitor visit:<br>**Pilot**: An on-site visit per site* after 2 or 3 participants concluded the study. In consultation with the client, this agreement can be adapted if, for example, the rate of inclusion is strong at one site and weak at the other.<br>**Main Study**: After source data verification can be conducted for at least 10 participants.<br>Also in this case, in consultation with the client, the visits can be re-arranged to adjust for eventual skewedness in inclusion rates between the two sites.<br><br><u>Remote monitoring visits</u><br>Monitor will approach the study team by telephone/video conferencing. The appointment will be planned in advance, enabling the contact(s) to take action accordingly beforehand in case this is necessary. A monitor report will be sent to the principal investigator UMCG and action list to the study team.<br><br>* assuming the second site will start before conclusion of the pilot.
**Patient flow**	During each monitor visit:<br>- Check compliance with contract / assumed recruitment rate<br>- Specify reasons for non-compliance<br>- Discuss actions needed<br>- Check dropout rate (lost to follow-up, withdrawn ICF, follow-up not 'per protocol')
**For the following items check 50 % of the patients:**	
**Informed consent**	Check availability of signed informed consent form.
**For the following items check 25 % of the patients:**	
**Informed consent**	Check informed consent process fully.
**In- and exclusion criteria**	Check all inclusion and exclusion criteria.
**Source data verification**	Selective source data verification of the following items:<br>- primary endpoint<br>**Pilot**<br>Investigate the feasibility of the KETA-study design:<br>- ketamine administration does not lead to systolic and diastolic blood pressure above 200 and 120 respectively<br>- ketamine administration does not lead to excessive

	sedation as reflected by Richmond Sedation Score lower than -3<br>- ketamine administration does not lead to CADSS scores above 60<br>- informed consent time interval<br>- Questionnaires number and administration's order is feasible<br>- feasibility of the proposed blood sampling time points<br>- BSSI score (inclusion criterion of minimum 7 points not too low)<br>- Recruitment and consent rates satisfy expectations<br>- Enough (n = 1 out of 2 of the UMCG participants OR 50 percent of the UMCG participants (n = 2 or 3)) participants are willing to have an MRI scan and given scan can be planned within one day.<br>**Main Study**<br>BBSI score<br><br>If time allows it, secondary endpoints will also be monitored:<br>MADRS, CGI, SAFTEE, CADSS scores<br>Concentration scores of BDNF, fatty acids, ketamine<br>MRI data relative to frontolimbic and hippocampal areas and glutamate levels
**SAEs, SUSARs**	- Check reporting SAE/SUSAR process and templates/forms used for reporting: 25% of total amount of reported SAEs/SUSARs<br>- Check for any unreported SAEs/SUSARs of the patients that are verified (25% of patients)<br>- This includes reports in Toetsing Online and initial reporting + follow-up reporting
**Endpoints**	-Check for unreported endpoints<br>-Check process reporting endpoints
**Protocol compliance**	Check documentation and reporting of any noncompliances to protocol, GCP, WMO, WBP, GDPR, "geneesmiddelenwet" any other applicable regulation.
**Investigational Medicinal Product / Treatment / ~~Medical Device~~**	- Visit pharmacy<br>- Check availability and the version of instructions to patients<br>- Check procedures (responsibilities, conduct and documentation)<br>- Check availability drug accountability (patient level and site level)<br>- Check transportation logs<br>- Check temperature logs.<br>**<u>NOTE</u>: This is <u>not</u> a medical device study. This is stated in the document 'Kwalificatie definitie medische hulpmiddel.docx' also present in the trial-master file.**
**Randomisation**	**Pilot**: NA, all participants receive ketamine<br>**Main study:**<br>- Check availability and the version of instructions/procedures<br>- Check randomization and deblinding procedure<br>- Check emergency envelopes / blinding maintained

Section 7: Specifications of monitoring visit continued	
**For the following items perform 1 checks per site:**	
**Laboratory**	- Facilities: access, labels, shipment materials, freezer, temperature monitor, calibration/use/maintenance/documentation of equipment<br>- Certification: ISO certification<br>- Procedures: presence of SOPs and execution of BDNF analysis
**Investigator Site File / Trial Master File**	- Check completeness file using Table of Contents<br>- Check correct storage of file<br>- Check correct storage of source document
**Logistics of data and database**	- Check logistics of CRFs (sending electronic data, in multicenter studies copy on site)<br>- Check procedure of data flow and query handling (data entry, checks, queries data manager, queries monitor, data correction, special attention to process in multicenter studies)<br>- Check procedures related to database (access, back-up procedure, security, privacy, lock, saving and archiving, version control, audit trail)
**Site staff**	- Check of qualifications (CV's and training log)<br>- Check of delegation log (completeness, content)

In case of noncompliance related to safety, discuss finding with the Principal Investigator and decide whether extra monitoring visits are indicated to check a larger sample of patients. This may result in more monitoring visits than specified above.
